# A Review of Rechargeable Zinc–Air Batteries: Recent Progress and Future Perspectives

**DOI:** 10.1007/s40820-024-01328-1

**Published:** 2024-02-29

**Authors:** Ghazanfar Nazir, Adeela Rehman, Jong-Hoon Lee, Choong-Hee Kim, Jagadis Gautam, Kwang Heo, Sajjad Hussain, Muhammad Ikram, Abeer A. AlObaid, Seul-Yi Lee, Soo-Jin Park

**Affiliations:** 1 Department of Nanotechnology and Advanced Materials Engineering, Hybrid Materials Research Center (HMC), Sejong University, Seoul, 05006 Republic of Korea; 2 Department of Chemical and Biomolecular Engineering, Yonsei University, 50 Yonsei-Ro, Seodaemun-Gu, Seoul, 03722 Republic of Korea; 3 Department of Chemistry, Inha University, Incheon, 22212 Republic of Korea; 4 Solar Cell Applications Research Lab, Department of Physics, Government College University Lahore, Lahore 54000 Punjab, Pakistan; 5 Department of Chemistry, College of Science, King Saud University, P.O. Box 2455, Riyadh, 11451 Saudi Arabia

**Keywords:** Zinc–air batteries, Energy storage, Affordability, Reversibility

## Abstract

Zinc–air batteries (ZABs) are gaining attention as an ideal option for various applications requiring high-capacity batteries, such as portable electronics, electric vehicles, and renewable energy storage. ZABs offer advantages such as low environmental impact, enhanced safety compared to Li-ion batteries, and cost-effectiveness due to the abundance of zinc. However, early research faced challenges due to parasitic reactions at the zinc anode and slow oxygen redox kinetics. Recent advancements in restructuring the anode, utilizing alternative electrolytes, and developing bifunctional oxygen catalysts have significantly improved ZABs. Scientists have achieved battery reversibility over thousands of cycles, introduced new electrolytes, and achieved energy efficiency records surpassing 70%. Despite these achievements, there are challenges related to lower power density, shorter lifespan, and air electrode corrosion leading to performance degradation. This review paper discusses different battery configurations, and reaction mechanisms for electrically and mechanically rechargeable ZABs, and proposes remedies to enhance overall battery performance. The paper also explores recent advancements, applications, and the future prospects of electrically/mechanically rechargeable ZABs.

## Highlights

- Recent progress in Zn–air batteries is critically reviewed.
- Current challenges of rechargeable Zn–air batteries are highlighted.
- Strategies for the advancement of the anode, electrolyte, and oxygen catalyst are discussed.
- Future research directions are provided to design commercial Zn–air batteries.

## Graphical abstract

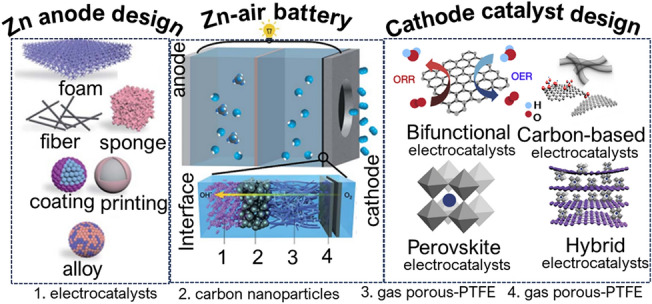

## Introduction

**Fig. 1:**
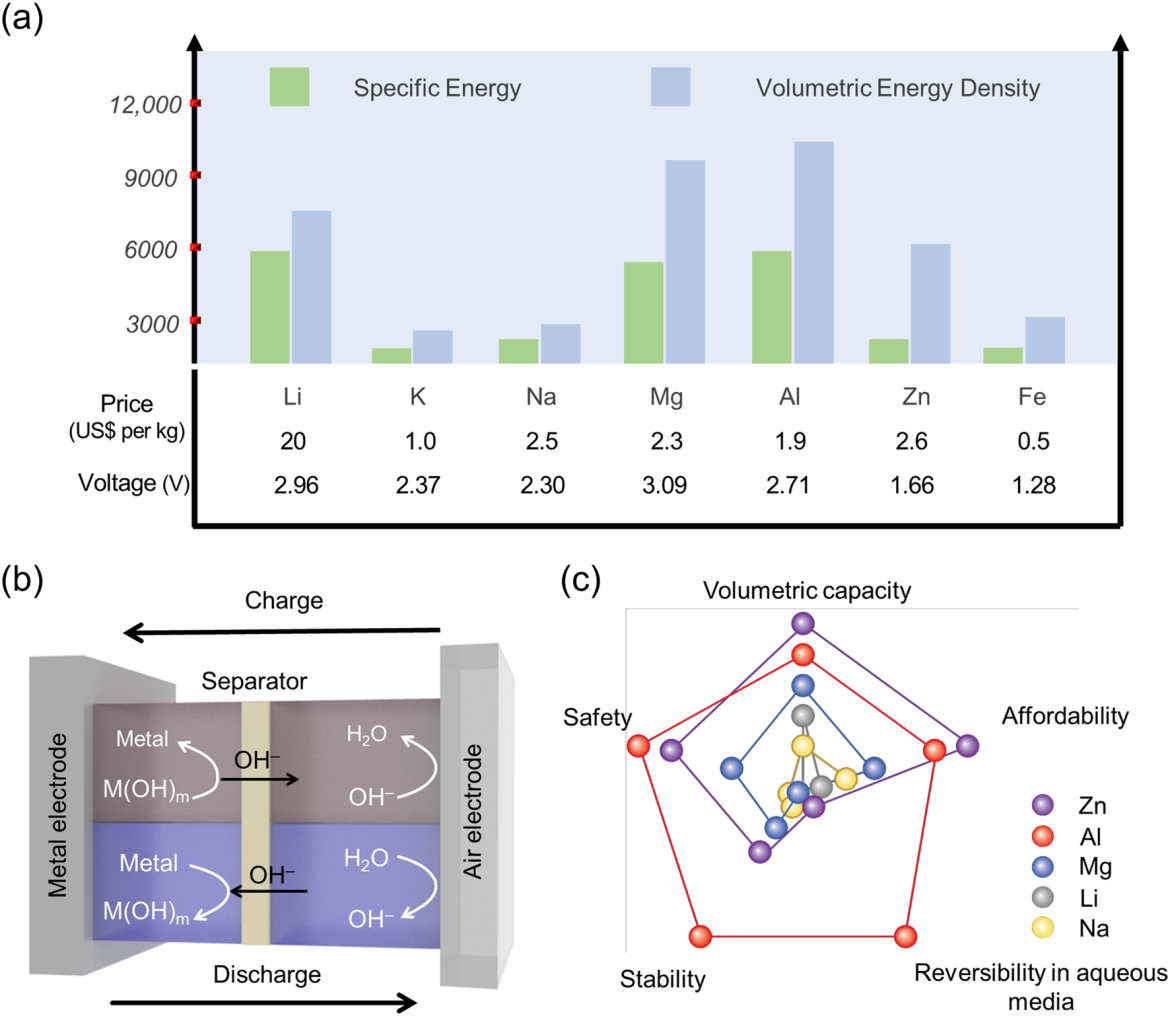
**a** Theoretical specific energies, volumetric energy densities, nominal cell voltages, and properties for various metal anodes, **b** schematic diagram of a ZAB, and **c** comparison of the theoretical specific energies, safety, stability, reversibility in aqueous media, and affordability of metal–air batteries [22, 23]

The intermittent nature of renewable energy sources necessitates efficient energy storage solutions. This has spurred research and widespread adoption of renewable energy systems, including the development of rechargeable batteries driven by rising demand for electric vehicles (EVs) and Internet of Things (IoT) sensors [1, 2]. At present, lithium-ion batteries (LIBs) are the leading rechargeable battery technology available in commercial applications, including EVs, portable electronics, and medical devices [3, 4]. However, researchers are seeking alternatives to LIBs due to their high cost, low energy density, and toxicity [5]. In contrast to other metals, zinc (Zn) boasts a notably lower price ($2.6 per kg) compared to lithium (Li) ($20 per kg), along with a compact ion radius (0.74 Å) that complements its high energy density. The superior features of ZABs relative to other batteries are illustrated in Fig. 1. Zinc–air batteries (ZABs) have garnered attention as a promising alternative due to their compelling attributes, including impressive theoretical energy densities of 1218 Wh kg^−1^ (gravimetric) and 6136 Wh L^−1^ (volumetric) [6, 7], eco-friendliness of harnessing power from Zn and atmospheric oxygen, and their compact form factor attributed to the air cathode, and significantly low operating cost of < $10 kW^−1^ h^−1^ [8]. Historically, primary ZABs found commercial use in the nineteenth century for hearing, navigation, medical, and railroad signal applications, owing to their high energy density. Furthermore, zinc's inherent low reactivity and robust stability in aqueous electrolytes position it for sustained cyclic operation in rechargeable applications [9–11]. In fact, prior to the ubiquity of LIBs, ZABs were proposed as the future power source for electric vehicles (EVs) [7, 10, 12].

In the past few decades, substantial progress in the development of electrode materials, electrolytes, interfacial science, and model designs of rechargeable ZABs. However, achieving stable electrochemical operation is the main obstacle for the commercialization of rechargeable ZABs. The fundamental challenges such as high polarization and fast degradation of the air cathode, low interfacial compatibility and stability of the electrolyte, and poor electrochemical irreversibility of the Zn anode must be resolved to get stable operation of rechargeable ZABs [1, 13–16]. Thus, the rational design of individual components and device systems is essential to achieve a satisfactory performance in sustainable ZABs. The deeper understanding of the electrocatalytic oxygen reduction/evolution reaction (ORR/OER) of the air cathode, electrolyte, Zn anode, and electrolyte/electrode interface stability is needed as it exerts a great influence on the reaction kinetics, performance, etc., of rechargeable ZABs. In addition, maintaining the electrochemical interface stability is the key to ensuring the long-term stable operation of ZABs [17–21].

Many studies have highlighted the potential of ZABs as a promising alternative to conventional rechargeable batteries with assessments covering the entire system or specific components [24–27]. However, to shed light on recent research activities, it is crucial to provide an overview of the current progress and remaining challenges for state-of-the-art ZABs especially with respect to the design principles of key materials and their structure–property relationship at the atomic level. In recognition of this, we aim to provide an up-to-date overview of the rapidly evolving field of ZABs issues such as Zn anode stabilization, oxygen electrocatalysts design, relevant reaction mechanisms, interface design of air cathodes, and electrolyte/electrode interfacial behaviors. Additionally, we provide a brief correlation of Zn electrode, electrolyte, separator, and air electrode for both primary and rechargeable ZABs, along with their configuration and operation. We comprehensively discuss new concepts of electrocatalysts such as perovskite oxide-based, carbon-based, and hybrid or mixed for mechanically rechargeable ZABs, and flexible ZABs. This review then concludes with a summary and future directions for ZAB development.

## Fundamentals, Working Principle, and Mechanism of Rechargeable ZABs

**Fig. 2:**
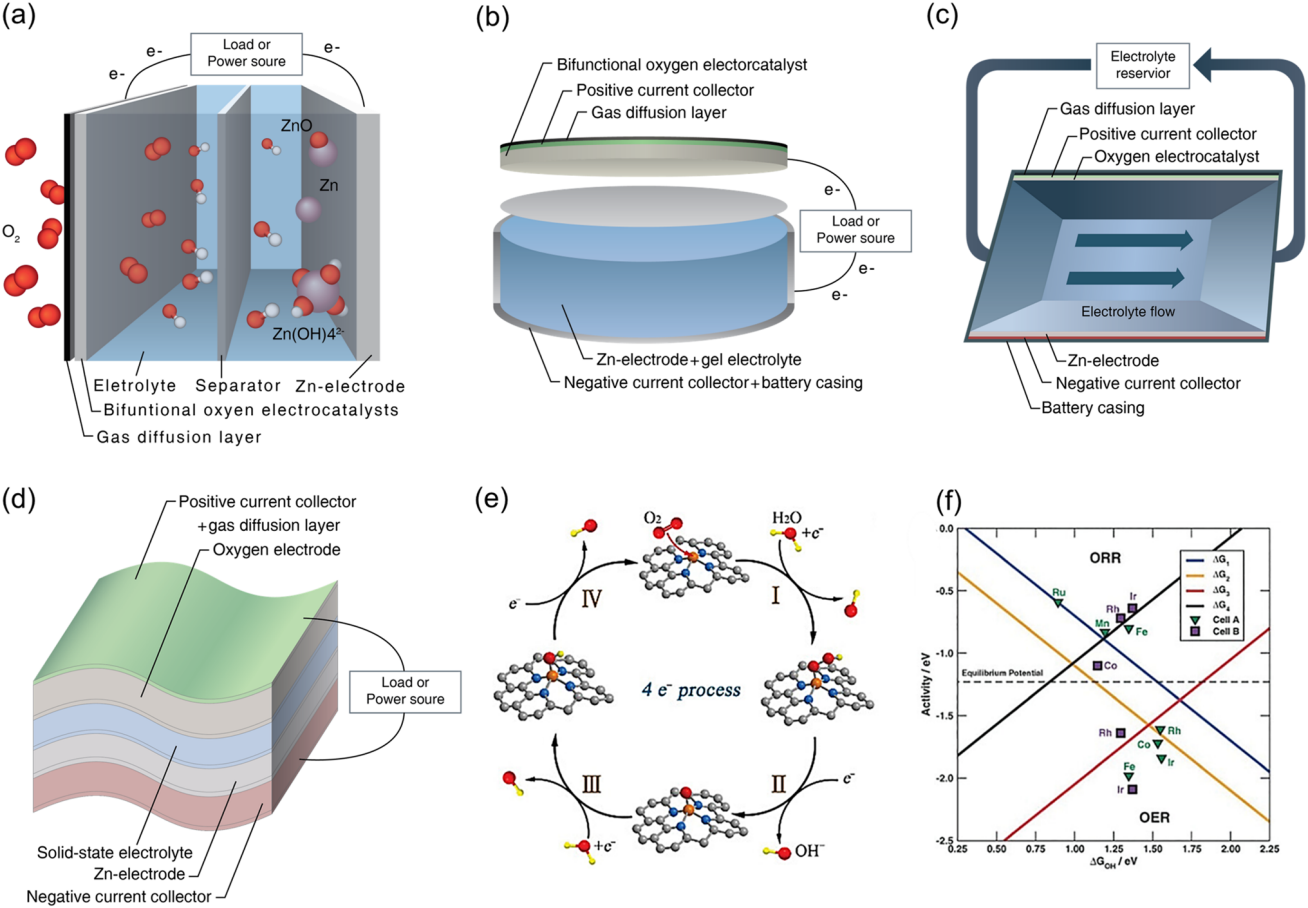
Four common rechargeable ZAB configurations: **a** a planar battery with an aqueous electrolyte, **b** a planar battery with a gel electrolyte, **c** a flow battery, and **d** a flexible battery. Reproduced with permission from [28]. **e** Illustration of intermediates in the ORR and OER processes. **f** Theoretical ORR and OER volcano plots of overpotential based on the scaling relationships [50]. Copyright 2023, Wiley

The various forms of electrically rechargeable ZABs are illustrated in Fig. 2. The conventional planar configuration was designed to achieve a high energy density, while flow batteries for realizing high cycle numbers and operational lifetimes. Flexible ZABs are particularly important for portable electronic devices needing high energy–density and flexible designs [28]. Figure 2a illustrates a usual planar configuration of rechargeable ZAB consisting of four essential components: (i) a Zn electrode; (ii) an air electrode consisting of a gas diffusion layer (GDL), current collector, and bifunctional oxygen electrocatalyst; (iii) a liquid electrolyte; and (iv) a separator. In the laboratory, plastic plates, chambers, and gaskets are widely used to simplify the battery assembly. In Fig. 2b, gel electrolytes are used in place of liquid electrolytes in planar batteries. Typically, these gel electrolytes are based on conductive hydrogels.

Figure 2c is commonly used rechargeable ZABs with a liquid electrolyte reservoir between the Zn and air electrodes that is refilled to improve runtime and battery life. Refreshing the electrolyte can slow the deterioration of Zn electrode and eliminate carbonate precipitates in air electrode [29, 30]. Figure 2d shows a flexible battery arrangement suitable for portable systems [31–34]. The planar electrodes and solid-state electrolyte can bend and twist without damage [35, 36]. These can also take on different shapes including a coaxial cable design for flexible batteries [37, 38].

The working principles of primary ZABs remain the same across the different configurations. A conventional ZAB is composed of an alkaline electrolyte, a negative Zn electrode, a membrane separator, and a positive air electrode as shown in Fig. 2a. Oxidation of Zn produces soluble zincate ions (Zn(OH)_4_^2−^) during battery discharge, which then transform into insoluble zinc oxide when supersaturated in the electrolyte [12, 39]. The reactions are as follows:

Negative Zn electrode reactions:

1 $${\text{Zn}} + 4{\text{O}}{{\text{H}}^- } \to {\text{Zn}}\left( {{\text{OH}}} \right)_4^{2 - } + 2{{\text{e}}^- }$$

2 $${\text{Zn}}\left( {{\text{OH}}} \right)_4^{2 - } \to {\text{ZnO}} + {{\text{H}}_2}{\text{O}} + 2{\text{O}}{{\text{H}}^- }$$

Positive air electrode reaction:

3 $${{\text{O}}_2} + 4{{\text{e}}^- } + 2{{\text{H}}_2}{\text{O}}\; \to 4{\text{O}}{{\text{H}}^- }$$

Overall reaction:

4 $$2{\text{Zn}} + {{\text{O}}_2} \to 2{\text{ZnO}}$$

Parasitic reaction in the Zn electrode**:**

5 $${\text{Zn}} + 2{{\text{H}}_2}{\text{O}} \to {\text{Zn}}{\left( {{\text{OH}}} \right)_2} + {{\text{H}}_2}$$

Poisoning of the electrolyte:

6 $${\text{KOH}} + {\text{C}}{{\text{O}}_2}\; \to {\text{KHC}}{{\text{O}}_3}\;{\text{or}}\;\;2{\text{KOH}} + {\text{C}}{{\text{O}}_2}\; \to {{\text{K}}_2}{\text{C}}{{\text{O}}_3} + \;{{\text{H}}_2}{\text{O}}$$

When Zn and water react, hydrogen gas production as well as oxidation occurs on the negative electrode. This phenomenon is called a parasitic reaction, which results in gradual corrosion and efficiency reduction of Zn metal. Oxygen in the air diffuses via the porous gas diffusion electrode, which acts as a positive electrode, where it is reduced on the electrocatalyst surface when it comes into direct contact with the electrolyte [40]. Interestingly, at the air electrode of ZABs, hydroxide ions are the main product of the ORR. This process is analogous to the ORR that occurs in alkaline hydrogen fuel cells [41, 42], and in fact the electrode design and catalyst materials for these energy conversion systems are identical. As such, several interesting ORR catalysts have been used in fuel cells that could also be applied to ZABs [43–46].

Recharging ZABs involves reversible electrochemical processes, resulting in Zn metal plating on the negative electrode and oxygen evolving from the positive electrode. Zn is highly active and can be plated in an aqueous electrolyte, but the discharge product zincate is highly soluble and often escapes the negative electrode area, leading to low cyclability. An incomplete reversal of zincate during recharging can cause electrode shape changes and dendrite formation, which can reduce battery performance or even cause a short circuit [47, 48]. Zn metal electrodes are also concerned with parasitic hydrogen evolution reaction (HER)-mediated corrosion and uneven deposition of zincate ion (Eq. 5). These issues can lead to reduced cycling stability of the cell or even a short circuit if Zn dendrites penetrate the battery separators. In addition, the electrolyte could evaporate or be diluted with extended usage depending on the relative humidity of the surrounding environment. Alkaline electrolytes can react with ambient CO_2_ (Eq. 6), leading to salt precipitate formation that can eventually clog the porous framework of the air electrode [28, 39]. Thus, research efforts are necessary to develop electrolytes and optimize the interaction of electrolyte–electrodes aiming to improve the overall performance of ZABs. In contrast, the ORR and OER of the air cathode also affect equally the energy efficiency, power density, and durability of the rechargeable ZABs. Due to the complex multi-electron reaction process and slow kinetics of the ORR and OER processes, the energy efficiency of the battery is generally reduced to 55–65%, which poses a great challenge to the scalability of rechargeable ZABs. Therefore, development of high-performance bifunctional oxygen electrocatalysts by analyzing its reaction pathway is very important for the advancement of rechargeable ZABs [49]:

### Mechanisms of ORR

The cathodic ORR proceeds via two main pathways in alkaline electrolytes. In one type, bidentate oxygen molecules are adsorbed on the active site (*) and then undergo a direct four-electron (4e^−^) path to generate OH^−^, which is a widely accepted faster reaction pathway (Fig. 2e). The reaction steps are as follows [50]:

7 $$* + {{\text{O}}_2}\left( {\text{g}} \right) + {{\text{H}}_2}{\text{O }}\left( {\text{l}} \right) + {{\text{e}}^- } \to *{\text{OOH}} + {\text{O}}{{\text{H}}^- }\left( {{\text{aq}}.} \right)$$

8 $$*{\text{OOH}} + {{\text{e}}^- } \to *{\text{O}} + {\text{O}}{{\text{H}}^- }\left( {{\text{aq}}.} \right)$$

9 $$*{\text{O}} + {{\text{H}}_2}{\text{O}}\left( {\text{l}} \right) + {{\text{e}}^- } \to *{\text{OH}} + {\text{O}}{{\text{H}}^- }\left( {{\text{aq}}.} \right)$$

10 $$*{\text{OH}} + {{\text{e}}^- }\; \to * \, + {\text{O}}{{\text{H}}^- }\left( {{\text{aq}}.} \right)$$

The other involves two 2e^−^ pathways with the adsorption of O_2_ molecules at the top, producing H_2_O_2_ as an intermediate species. The ORR proceeding with this reaction pathway is relatively slow. The reaction equation is as follows.

11 $${{\text{O}}_2}\left( {\text{g}} \right) + {{\text{H}}_2}{\text{O}}\left( {\text{l}} \right) + 2{{\text{e}}^- } \to {\text{H}}{{\text{O}}_2}^- + {\text{O}}{{\text{H}}^- }\left( {{\text{aq}}.} \right)$$

12 $${\text{H}}{{\text{O}}_2}^- + {{\text{H}}_2}{\text{O }}\left( {\text{l}} \right) + 2{{\text{e}}^- } \to 3{\text{O}}{{\text{H}}^- }\left( {{\text{aq}}.} \right)$$

A clear understanding of the adsorption energy of oxygen intermediates at each reaction step is a prerequisite for searching ORR/OER bifunctional catalysts.

### Mechanisms of OER

The OER reaction pathway of ZABs is opposite to the four-electron path of the ORR (Fig. 2e). Recently, transition metal oxides such as rutile and tremolite have been extensively studied for OER, and the free energies of oxygen intermediates have been systematically investigated. The free energy and active site requirements of ORR and OER are different, making it difficult for a catalyst to exhibit two excellent electrochemical properties at the same time. A bifunctional volcano model built on the scaling relationship between the free energies of the oxygen intermediates in the ORR and OER processes is shown in Fig. 2f. In the volcano diagram, the ORR triangle undergoes the reduction steps of *OH (ΔG_1_) and O_2_ (ΔG_4_), while the OER region undergoes the generation of *OH (ΔG_2_) and *OOH (ΔG_3_). Therefore, the ΔG_OH_ values required to reach the summit of the ORR and OER volcanoes are not the same. Adjusting the ΔG_OH_ value of the catalyst to obtain optimal ORR activity will result in the loss of optimal OER activity and vice versa. Thus, maintaining the tops of the OER and ORR volcanoes as close to each other as possible according to the scaling relationship is pivotal for designing efficient bifunctional catalysts for ZABs [50, 51].

## Challenges and Progress on Basic Components of ZABs

ZABs technology has a long way to go to overcome the challenges and make it commercially viable. ZABs typically have a short lifetime due to the deactivation of the air catalyst during charging, resulting in low current density and high overpotential values. High potentials lead to oxidation and corrosion of the oxygen electrocatalyst. In addition, the porous structure of the air electrode is too fragile to withstand gas evolution (OER) during charging, leading to mechanical degradation of the electrode material and loss of activity, which ultimately leads to battery degradation. In addition, the formation of dendrites and unwanted parasitic reactions leading to HER (Eq. 5) significantly reduce the moisture level of the electrolyte, leading to corrosion of the zinc electrode and reduced battery life. Similarly, the electrolyte causes technical problems such as ionic conductivity, increased Zn solubility (reducing the available active surface of the Zn anode), and electrolyte evaporation, all of which limit the practical usability of rechargeable (ZAB) batteries. At the same time, atmospheric carbon dioxide CO_2_ interacts with electrolyte species to form carbonate deposits that interfere with ionic conductivity and block air diffusion pathways at the air electrode surface. Therefore, various research experiments need to be conducted to explore catalytically active materials, suitable electrode structures, electrolyte compositions, and Zn anode materials to improve the round-trip efficiency and power output of rechargeable ZABs [1, 52].

### Anode Materials

Improving the reversibility of the Zn anode and alleviating issues such as dendrite formation, passivation, hydrogen evolution, and corrosion have been critical areas of focus in enhancing the lifetime of ZABs. Zn electrodes, constructed from plates, foil, or compressed particles, have recently been replaced by three-dimensional (3D) porous structures to improve their surface area and inhibit dendrite formation through direct contact with the electrolyte. Parker et al*.* [53] achieved a Zn utilization rate of almost 90% in a primary ZAB using a 3D Zn electrode with interconnected Zn domains and a monolithic void space. Another study [54] compared compressed powder-type Zn with 3D Zn electrodes and found that ZnO deposition occurred uniformly within the void/space of the 3D Zn electrode, allowing it to be cycled more than 100 times at 40% discharging. However, it should be noted that the high surface area of 3D Zn electrodes may lead to corrosion and HER, reducing battery life.

**Fig. 3:**
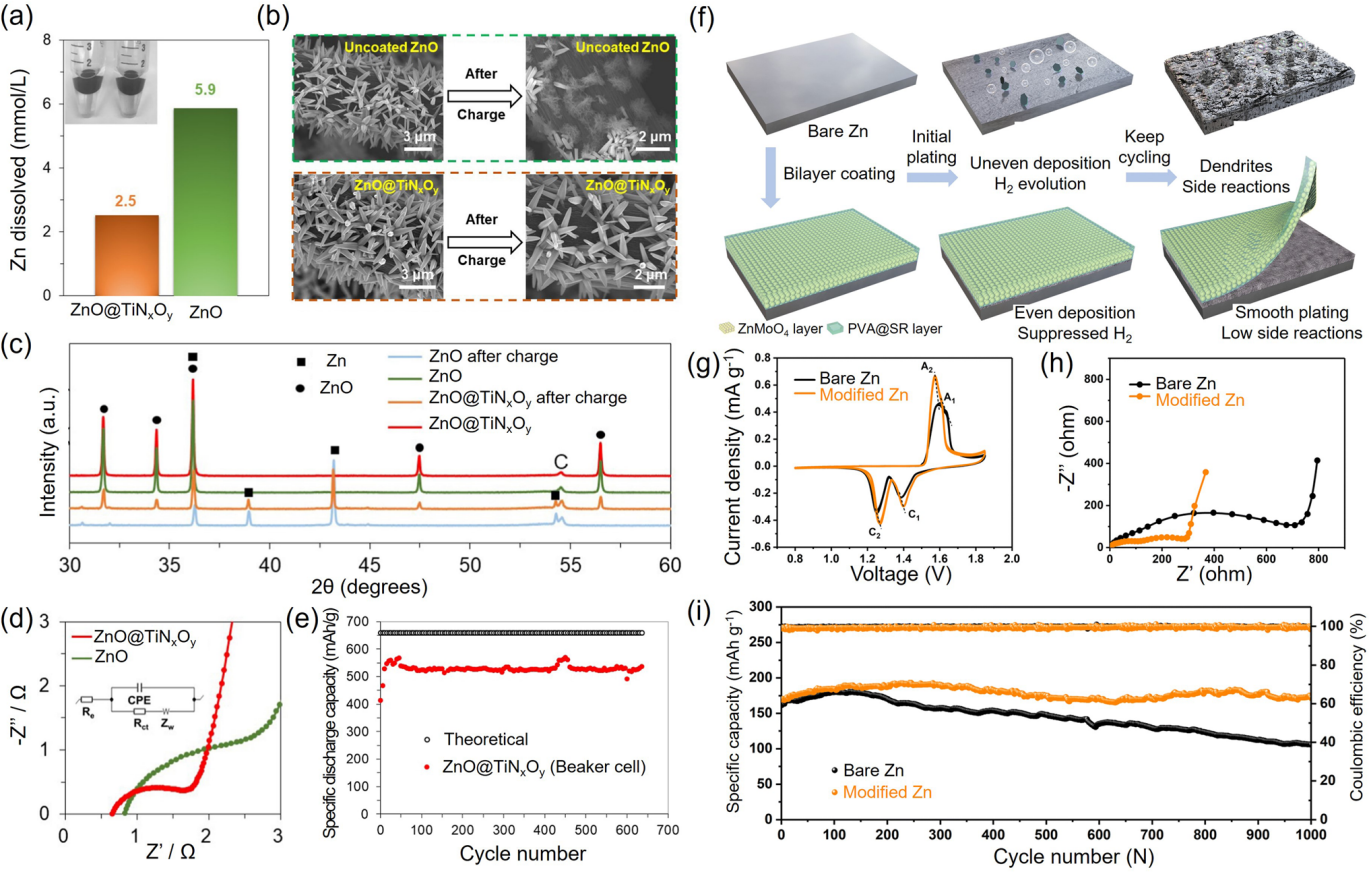
**a** Zn dissolution (mmol L^−1^) in a 4 M KOH solution. **b** Scanning electron microscopy (SEM) micrographs of uncoated ZnO and ZnO@TiN_x_O_y_ anode before and after the charging process. **c** X-ray diffraction patterns for ZnO nanorods and a ZnO@TiN_x_O_y_ anode before and after the charging process. **d** Electrochemical impedance spectroscopy (EIS) results and the related equivalent circuit for uncoated ZnO and a ZnO@TiN_x_O_y_ nanorod anode. Reproduced with permission from [64]. **e** Cycling performance of ZnO@TiN_x_O_y_ nanorod anode (2 mg cm^−2^) with 200 cycles ALD at 0.5 C charge and 2 C discharge rates in beaker cell with 10 mL ZnO saturated 4 M KOH electrolyte. The cutoff voltages are 1.4/2 V. One dot every five data points. Produced with permission from [64]. **f** Cycling performance of bare Zn and PVA@SR-ZnMoO4 SEI-like structure coating modified Zn. **g** CV curves at 0.1 mV s^−1^. **h** EIS curves before cycling. **i** Long-term cycling performance at 1.0 A g.^−1^. Produced with permission from [65]

Innovative designs based on coatings or composite design via alloying or chemical coating have emerged as effective strategies to improve the reversibility of Zn anode [55, 56]. Sun et al*.* utilized a simple displacement reaction to construct a homogeneous and densely structured Cu/Zn composite anode, which can be converted into a CuZn/Zn composite during cycling and improves the corrosion resistance of zinc anodes [57]. Chen et al*.* used in situ reduction and self-alloying processes to coat a CuxZny alloy layer on Zn foil anode, which can act as a nucleating agent to protect the growth of zinc dendrites resulting in enhanced electrochemical performance of ZABs [58]. Jo et al*.* [55] achieved 91.5% corrosion inhibition efficiency and 99.5% retention in discharge capacity of a primary ZAB using a Zn-Bi alloy. Aremu et al*.* [59] demonstrated dendrite-free cycling and high capacity without passivation in a secondary battery using a Zn anode coated with bismuth oxide, potassium sulfide, and Pb (II) oxide additives. In addition, metal coating using aluminum oxide (Al_2_O_3_), copper oxide (CuO), and titanium oxide (TiO), etc., have been found to improve the ZABs performance by forming a protective anodic layer and suppressing spontaneous side reactions [60–63]. Zhang et al*.* [64] synthesized ZnO@TiN_x_O_y_ core/shell nanorods using atomic layer deposition as an anode, where the TiN_x_O_y_ coating effectively reduced the dissolution of Zn, lowered the internal resistance and facilitated the charge transfer (Fig. 3a − e). The ZAB using thin TiN_x_O_y_ coating showed a stable discharge capacity of 508 mAh g^−1^ over 7500 cycles by efficiently blocking heavier zincate molecules while allowing OH^−^ and H_2_O molecules to permeate through the anode. Zhang et al. [65] fabricated PVA@SO_4_^2−^ receptor–ZnMoO_4_ with SEI-like structure coating to stabilize Zn surfaces (Fig. 3f). The SEI-like structure of SO_4_^2−^ receptor (SR) enhanced the dispersion of counterion Zn^2+^ to inhibit dendrite formation. And the inorganic ZnMoO_4_ as protective layer suppresses dendrites and side reactions. As shown in Fig. 3g, h, modified Zn cell indicated improvement of charge/discharge capacity compared with pristine Zn regarding smaller potential gap of redox peak representing smaller electrochemical polarization and higher reactivity and smaller semicircle representing rapid charge transfer. Moreover, the modified Zn cell represents over 90% specific capacity retention, whereas pristine Zn shows only 59% cyclic retention after 1000 cycles at 1 A g^−1^ (Fig. 3i).

Organic anode coatings are more cost-effective, easily fabricated, environmentally friendly, and controllable than inorganic coatings. Extensive research has been conducted on organic additives including polyvinyl alcohol (PVA), polyacrylonitrile (PAN), and polyaniline (PANI) hydrogels, inhibiting the self-discharge and corrosion of ZABs [66, 67]. Zhang et al*.* used a polymer binder to encapsulate Zn metal and create a stable 3D ZnO/PVA/β-CD/PEG composite electrode that significantly reduced anode distortion with > 80 cycles [68]. Moreover, organic additives can behave as impurities and insulators, improving battery impedance [63]. However, organic additives can reduce the specific energy of batteries and increase weight and cost. Consequently, it is necessary to select them carefully and optimize the anode composition.

Typically, the use of additives can reduce the specific energy of a battery and increase its weight and cost, thus it is essential to select additives carefully and optimize the anode composition. Inorganic and organic anode additives produce a protective layer similar to SEI that hinders electrolyte access and immobilizes zincate ions, thereby reducing parasitic anodic reactions [69, 70]. Despite some progress, there have been limited studies on anode additives, making it difficult to identify the mechanisms and effects of each additive, particularly with respect to specific materials. Thus, a more systematic analysis is needed to understand how different coating materials affect the degradation and battery stability.

The structural design and composition optimization of Zn electrodes has been extensively studied to achieve the excellent activity of ZABs. Morphology alteration of the anode is a strategy to enhance its activity and reduce deformation. An excessively porous structure can adversely affect electrode resistance and the corrosion rate, but fine-tuning can promote mass transport and electrochemical activity by increasing the surface area. Recently, researchers have reported that Zn anodes with a porous and sponge-like 3D architecture can be produced, which has the potential to increase their reversibility. Moreover, ZABs with sponge-like electrodes can be employed in wearable devices due to their compressibility and mechanical flexibility. Lin et al*.* demonstrated that electroplating a porous Zn anode at 500 Hz resulted in a doubled specific surface area and a 60% increase in power density [71]. In a study by Pan et al*.* [72], a sponge-like anode was formed by electrodeposition of Zn nanosheets on N-doped carbon foam, resulting in a high mechanical strength, a power density of 260 mW cm^−2^, and a low voltage gap of 0.657 V at 5 mA cm^−2^. Additionally, the eutectic-composition alloying, 3D printing, and gradient design is an effective strategy to substantially tackle side reaction and severe dendrite growth of zinc anode, leading to rapid capacity fading and short lifespan of rechargeable ZIBs. For instance, Wang *et.al.* reported the lamellar structure, composed of alternating zinc and aluminum nanolamellas, which delivered dendrite-free zinc stripping/plating for more than 2000 h, high energy density and 100% capacity retention of ZABs after 200 h of cycling [73]. Zhang *et.al* constructed 3D Ni–Zn anode with multi-channel lattice structure and super-hydrophilic surface by combining 3D printing and electroless plating/electroplating techniques which induced the uniform deposition of Zn without Zn dendrite growth and highly reversible Zn plating/stripping with satisfactory coulombic efficiency [74]. Cao *et.al.* reported an imprinted gradient zinc electrode that prohibited side reactions between the electrolyte and the zinc anode and suppressed dendrite growth. The resulting imprinted gradient zinc anode was cycled stably for 200 h at a high current density/capacity of 10 mA cm^−2^/10 mAh cm^−2^ by outperforming the none-gradient counterparts [75]. Thus, structural design and composition optimization strategy can be a promising way for dendrite-free ZABs at high current densities and high capacities. Table 1 summarizes recent optimization strategies for Zn anodes in ZABs.

**Table 1:** Zn anodes used for the construction of ZABs

Structural assessment and electrolyte	Anode	Specific capacity (mAh g^−1^)	Discharge/charge voltage gap (V)	Cyclic performance	References
Hard carbon (HC), 1 M Zn(OTf)_2_	rGO-SnCu/Zn	–	~ 0.7	1200 h @ 0.25 mA cm^−2^	[76]
Zn surface modification, 2 M ZnSO_4_ + 0.2 M MnSO_4_	Zn-AgNWs	243.9	0.76	800 cycles@0.6 A g^−1^	[61]
Bottom cell, 2 M Zn(SO_4_)_2_ + 0.1 M MnSO_4_	Zn@ZrP	132.4	–	780 h @ 0.5 mA cm^−2^	[77]
3D zinc anode, 1 M ZnSO_4_ + 1 M KCl	Ag-modified Cu foam	676	1.02	80 cycles, 2 h @ 25 mA cm^−2^	[78]
Coin cell, 3 M ZnSO_4_ + 0.1 M MnSO_4_	Zn-Sb_3_P_2_O_14_	111.7	–	450 h @ 10 mA cm^−2^	[79]
3D porous framework, 6 M KOH	Zn anode	812	0.63	33 cycles @ 5 mA cm^−2^	[80]
Coin cell, 6 M KOH and 0.2 M Zn(AC)_2_	Ti_3_C_2_T_x_-protected Zn	–	0.6	400 cycles @ 5 mA cm^−2^	[81]
Carbon cloth (CC) cathode, 2 M ZnSO_4_,	Zn@ZIF8	158	–	750 h @ 1.0 mA cm^−2^	[82]
Layered structure, 6 M KOH + 0.2 M Zn(Ac)_2_ + S ZnO aqueous solution	Tin-coated copper foam (CF@Sn)	800	–	5220 h @ 10 mA cm^−2^	[83]

### Electrolytes

ZABs usually use alkali electrolytes such as KOH and NaOH to optimize the activity of both air and Zn electrodes. KOH is preferred over NaOH for several reasons, including its high ionic conductivity, larger oxygen diffusion coefficient, and low viscosity [84]. Since carbonate precipitation is a major issue for ZABs, and the use of KOH can alleviate this by forming more soluble products (*e.g.,* K_2_CO_3_ or KHCO_3_) than their Na counterparts.

**Fig. 4:**
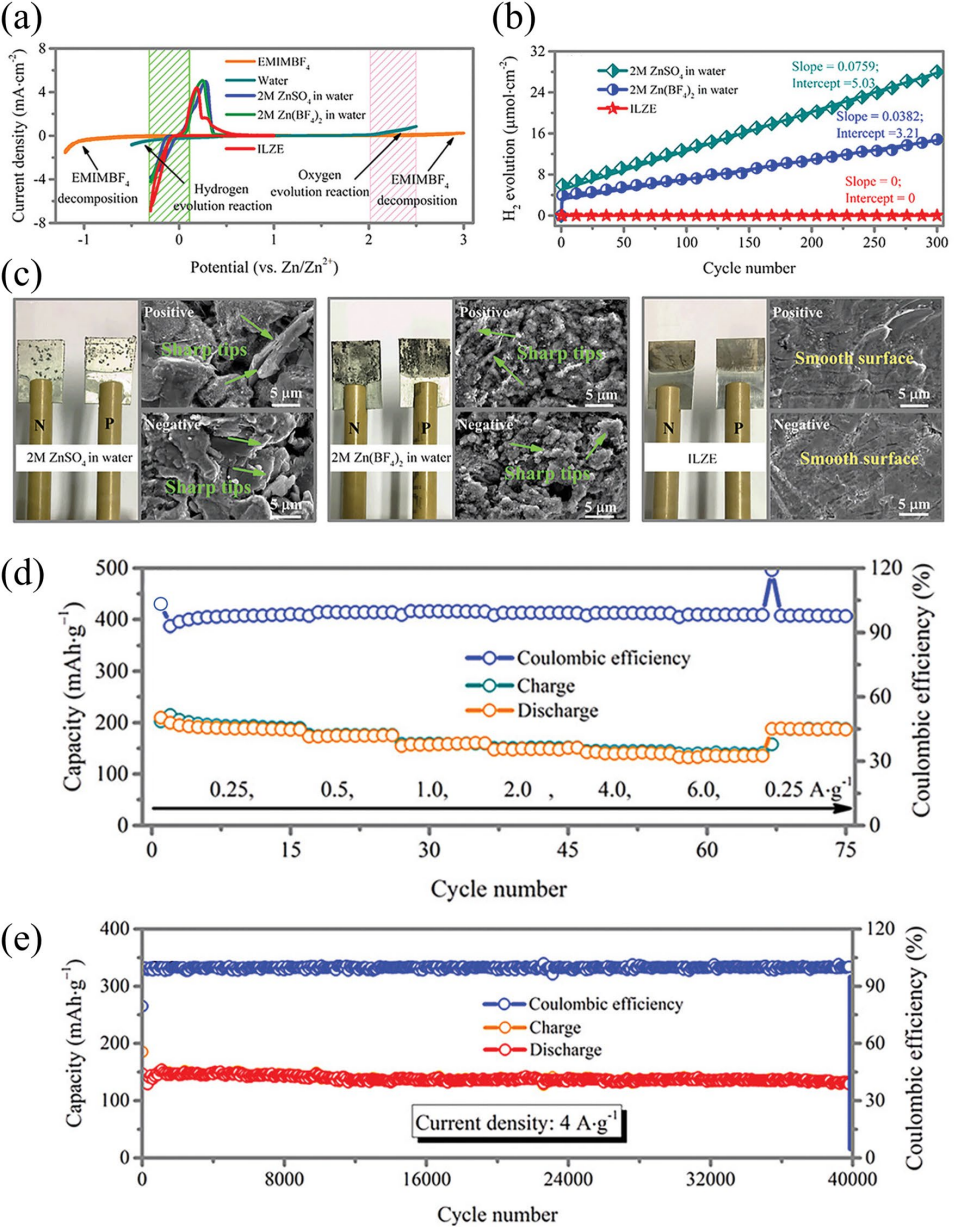
**a** Three-electrode configuration-based cyclic voltammetry (CV) curves for prepared Zn-ion battery in various electrolytes, **b** H_2_ evolution according to the number of cycles measured at 0.5 mA cm^−2^, **c** surface morphology of Zn foil after 300 cycles measured at 0.5 mA cm^−2^, **d** cyclic stability and coulombic efficiency at an applied current density of 4 A g^−1^ for 40,000 cycles, and **e** rate performance. Reproduced with permission from [90]

Significant advancements have been made in the development of alkaline electrolytes (Table 2), which are the most commonly used type in ZABs; however, Zn electrodes still face corrosion, surface passivation, and dendrite formation during cycling in ZABs [54]. The volatilization and toxicity of alkaline electrolytes due to CO_2_ also restrict the charge–discharge efficiency and lifespan of ZABs. To overcome these, acidic/neutral electrolytes are proposed to increase the reversibility of Zn anodes [85, 86]. However, these systems have drawbacks such as low coulombic efficiency due to the secondary reactions associated with Zn deposition and hydrogen evolution and the development of dendrites on the Zn anode, leading to a low cycling life and rapid discharging. Thus, finding effective and cost-effective electrolytes for practical applications is urgently needed. Non-aqueous ionic liquids (ILs) have shown great potential in this regard, as multiple studies have demonstrated their ability to eliminate ZAB dendrites and improve their electrochemical properties [87–89]. Ma et al*.* [90] reported ZABs with enhanced properties, including suppression of HER and Zn dendrites, using [EMIm]BF_4_-Zn(BF_4_)_2_ as the electrolyte and a cobalt hexacyanoferrate (CoHCF) cathode (Fig. 4a–c). The high ionic conductivity of IL-based electrolytes was responsible for the ultrahigh rate performance of the as-fabricated ZIBs, with a capacity retention of 98% (40,000 cycles) and an excellent coulombic efficiency of 100% without a loss of capacity (Fig. 4d, e).

**Table 2:** Summary of recently described electrolytes for ZABs

Electrolyte composition (type)	Electrode materials	Specific capacity (mAh g_zn_^−1^)	Power density	Cyclic performance	References
6 M KOH + 0.2 M zinc Acetate (alkaline)	Zinc plate//Co–Co_3_O_4_@NAC@NF	721 @10 mA cm^−2^	164 mW cm^−2^ @0.63 V	35 h@10 mA cm^−2^	[97]
6 M KOH + 0.2 M zinc Acetate (alkaline)	Zinc foil//Co_3_O_4−x_@CP	800 @5 mA cm^−2^	122 mW cm^−2^ @230 mA cm^−2^	150 h@5 mA cm^−2^	[98]
7 M KOH + 5–20% v/v DMSO (alkaline)	Zinc granules//MnO_2_@NF	550 @10 mA cm^−2^	130 mW cm^−2^ @150 mA cm^−2^	600 cycles@discharge @75 mA cm^−2^	[99]
6 M KOH + 0.2 M ZnCl_2_ (alkaline)	Zinc plate//Pt–SCFP@CC	781 @10 mA cm^−2^	122 mW cm^−2^ @214 mA cm^−2^	80 h@5 mA cm^−2^	[100]
8 M KOH + 0–50% v/v Ethanol (alkaline)	Zinc granules//MnO_2_@NF	470 @25 mA cm^−2^	32 mW cm^−2^ @30 mA cm^−2^	–	[101]
6.0 M KOH + 0.2 M Zn(OAc)2 polyacrylamide/montmorillonite (PAM/MMT) (GPE)	ZAB-Mn-SAC	631	30 mW cm^−2^	29 h@2.0 mA cm^−2^	[102]
KOH + DMSO + poly(2-acrylamido-2-methylpropanesulfonic acid)/polyacrylamide (PAMPS/PAAm) (GPE)	Organohydrogel electrolyte (OHE)-based ZAB	562	21.8 mW cm^−2^	45 h@2.0 mA cm^−2^	[103]
Polyacrylic acid (PAA) + polyacrylamide (PAM) in glycerol (GPE)	Zn anode// carbon cloth cathode containing Pt/C and RuO_2_	506.2	8.2 mW cm^−2^	10 h @ 1.0 mA cm^−2^	[104]
PAM-CNF/KOH/KI (GPE)	cable-type ZAB	743	10 mW cm^−2^	45 h @ 2 mA cm^−2^	[105]
poly(2-acrylamido-2-methylpropanesulfonic acid potassium salt) (PAMPS-K) + methyl cellulose (MC) (GPE)	Zinc plate // Co_3_O_4_ nanoparticle/CC	754.2	54.2 mW cm^−2^	24 h @ 1 mA cm^−2^	[106]

*NAC* N-doped active carbon, *NF* Nickel foam, *CP* Carbon paper, *CC* Carbon cloth, *DMSO* Dimethyl sulfoxide

The rapid innovation of wearable devices has increased the demand for reliable, flexible, and stretchable energy systems. Metal-air batteries, particularly highly safe ZABs, have attracted significant research interest due to their large specific and volumetric energy densities. However, their performance significantly deteriorates below 0 °C due to their lower electrolytic ionic conductivity and slow kinetics related to the ORR and OER on the air cathode surface during the charge–discharge processes. Thus, gel polymer electrolytes (GPEs) hold promises as a viable option to enhance ion transport in ZABs, thereby improving their electrochemical performance even in low temperatures (Table 2). Additionally, GPE can suppress the growth of Zn dendrites and the solubilization of active ingredients. These electrolytes prevent liquid leakage and simplify the production of ZABs by combining the functions of the separator and electrolyte into a single component.

**Fig. 5:**
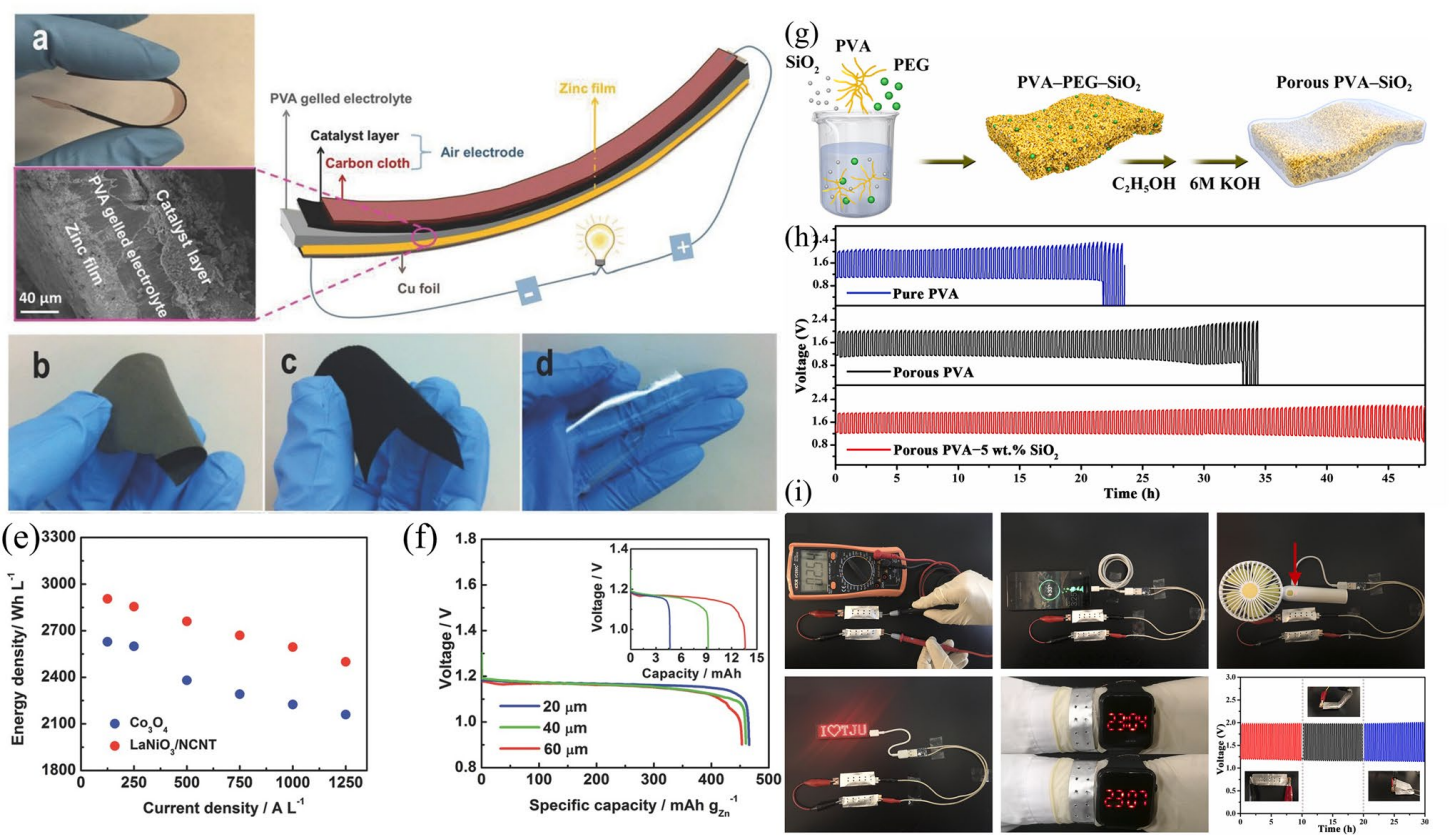
**a** Flexible, solid-state rechargeable ZAB illustrated using a photograph of its bending ability (top), a cross-sectional SEM image (bottom), and a schematic diagram of its structure (right). **b** Optical photograph of the freestanding Zn electrode film. **c** Optical photograph of the bifunctional catalytic air electrode using LaNiO_3_/NCNT. **d** Optical photograph of porous PVA-gelled electrolyte membrane. **e** Comparative analysis of the energy and current density for an all-solid-state ZAB prepared using the bifunctional catalyst Co_3_O_4_ and a LaNiO_3_/NCNT-based air electrode. **f** Specific capacity curves for the prepared ZABs as a function of the Zn film thickness. Reproduced with permission from [95]. **g** Scheme displaying the preparation of flexible a ZAB using a porous PVA nanocomposite-based GPE. **h** GCD curves for ZABs using different electrolytes at 3 mA cm^−3^ and 20 min per cycle. **i** Assembled ZABs used as a power source for various electronic devices. Reproduced with permission from [96]

Water loss is another problem of performance degradation in open system ZABs, and frequent addition of water is inevitable. GPE has been found to reduce water loss, improving the battery’s capacity and service life. Hydroponic gel, which can hold 20–100 times its weight in liquid, was investigated by Othman et al. as a gelling agent to immobilize KOH electrolytes for ZABs [91]. Subsequently, Mohamad et al. produced a battery containing 6 M KOH/hydroponic gel with a specific capacity of 657.5 mAh g^−1^ (789 W kg^−1^) [92]. Yang et al*.* [93] produced a GPE using polyethylene oxide (PEO) and PVA with suitable ionic conductivity, electrochemical stability, and mechanical strength for solid-state ZABs. Similarly, Zhu et al*.* [94] synthesized a GPE at room temperature by polymerizing acrylate, KOH, and H_2_O, which had a specific conductivity of 0.288 S cm^−1^. This GPE was successfully used in the laboratory to achieve performance nearly identical to that of an aqueous alkaline solution in Zn–air, Zn–MnO_2_, and Ni–Cd batteries. Fu et al*.* [95] developed a rechargeable, flexible ZAB using a GPE and prefabricated battery components of different sizes and shapes to meet the requirements of various applications. An optical photograph, a diagram of the entire device, and a cross-sectional scanning electron microscopy (SEM) image are displayed in Fig. 5a. To create the battery, a PVA-gelled electrolyte was laminated between the air electrode, which consisted of a bifunctional catalyst-loaded carbon cloth, and the Zn film electrode. The battery contained a Zn electrode film (Fig. 5b), a bifunctional catalytic air electrode (Fig. 5c), and a porous PVA-gelled electrolyte membrane (Fig. 5d). To test the battery performance, a LaNiO_3_/NCNT composite was used to measure the energy density with the current density (Fig. 5e). The cell using the LaNiO_3_/NCNT composite demonstrated a considerably higher energy density than that containing Co_3_O_4_ nanoparticles (NPs) due to the synergistic behaviors of LaNiO_3_ and NCNT, resulting in higher catalytic activity. This approach is solid-state, versatile, and simplifies ZAB production compared to aqueous electrolytes. However, challenges remain, such as low mechanical strength, high impedance at the electrode–electrolyte interface, and low ionic conductivity.

Initially, increasing the thickness of Zn film in the battery would pose issues, including reduced contact between Zn particles and electrolytes, increased diffusion resistance, and battery polarization, resulting in a loss in energy density. However, as indicated in Fig. 5f, batteries with varying Zn film thicknesses exhibited similar potential and specific capacity measurements until around 80% of a full discharge. Batteries with denser Zn films experienced a minor capacity loss in voltage reduction when completely discharged due to increased production of zinc oxide, which raises the Zn film’s internal resistance. Nevertheless, the 60-µm-thickness Zn film battery provided a capacity almost three times higher than that of the 20-µm due to the collaborative action of the gelled-PVA membrane and 3D Zn electrode in increasing Zn electrode accessibility to the electrolyte, which results in a weaker diffusion polarization and stable specific capacity (Fig. 5f, inset). The proposed battery exhibited a high energy density of 2905 Wh L^−1^, indicating its potential as an alternative to commercially available bendable Zn-MnO_2_ batteries and LIBs.

Fan et al*.* [96] reported a flexible ZAB with a high ionic conductivity GPE and enhanced electrolyte retention. Figure 5g depicts the manufacturing process of the porous PVA-based nanocomposite GPE. First, a polymer membrane was obtained by gelling a mixture of PVA, polyethylene glycol (PEG), and SiO_2_ powder (0, 1, 5, or 9 wt%), after which the pore-inducing agent was dissolved in ethanol. The obtained polymer membrane was then immersed in a highly alkaline KOH (6 M) solution. Next, the laminated structure of the ZAB was created using Zn foil and an electrocatalyst-loaded air electrode. The practical performance of the as-prepared GPE was tested by constructing a flexible ZAB configuration in which the GPE was placed between commercially available Co_3_O_4_-loaded carbon cloth and Zn foil. Figure 5h presents the galvanostatic charge–discharge (GCD) curves for three ZAB solid-state devices containing pure PVA, porous PVA, and the GPE as electrolytes. The ZAB with the highly conductive GPE (with porous PVA and 5 wt% SiO_2_) exhibited superior rechargeability, with cycling stability over 144 cycles (48 h). For this performance, two pre-assembled ZABs were connected in series, enclosed in aluminum plastic films with ventilation holes (Fig. 5i). The two ZABs connected as a power source to a mobile phone, electric fan, LED screen, and LED watch, achieving a high open-circuit voltage (OCV) of 2.54 V. The ZABs also showed high stability without significant potential change under various bending angles.

### Separators

ZABs utilize separators as physical barriers to ensure a safe distance between the Zn and air electrodes. They must be electrically resistant, highly conductive to ions, and electrochemically stable within the potential operating window. They also need to be mechanically robust to prevent short circuits caused by Zn dendrites. Porous polymer films such as polyethylene (PE), polyamide, and polypropylene (PP) have been used as separators in ZABs due to their ability to retain electrolytes while allowing for rapid OH^−^ transport. However, these separators have many drawbacks in liquid electrolytes such as electrolyte evaporation, severe Zn corrosion, high zincate crossover, and zinc dendrite formation, leading to a decline in efficiency of ZABs [107]. The surface functionalization strategies such as surface treatment with cationic or nonionic surfactants, sulfonation are effective in improving the hydrophilicity, electrolyte uptake capacity, and the OH^−^ transport. For example, sulfonated non-woven PP/PE separators exhibited higher hydrophilicity, resulting in double ionic conductivity when used in alkaline electrolytes [108, 109]. ZABs prepared using sulfonated separators have also demonstrated a power density of 27–38 mW cm^−2^. Inorganic polymer-based separators exhibit superior thermal stability compared with the organic counterpart. Saputra et al*.* [110] developed a ZAB by dip-coating the Zn electrode in a 5-μm MCM-41 membrane. The device structure was further enhanced by incorporating a commercially available air electrode in KOH. The prepared ZAB exhibited a power density of 32 mW cm^−2^ with a remarkable energy density of 300 Wh L^−1^. Its performance was found to be comparable with commercially available Zn–air button cells of similar sizes. Other approaches to consider involve utilizing commercially available PP membrane coatings that incorporate a copolymer synthesized from two IL monomers, allowing for anionic exchange [111]. Alternatively, impregnating Nafion with agents that repel anions has also been explored [112]. While modified separators offer extended battery life, the widespread adoption is hindered by the expensive nature of Nafion and ILs. To enhance ionic conductivity and ion selectivity of separators, more cost-effective approached are needed.

### Air Electrodes

An air electrode consists of three key components: an active layer, a GDL, and a current collector. Commonly, porous carbon materials combined with a wet-proofing binder such as polytetrafluoroethylene (PTFE) are used as the GDL [39]. The catalytic active layer, responsible for both the ORR and OER, consists of carbon materials, bifunctional catalysts, and the binder [113]. The GDL acts as an oxygen channel, providing a large hydrophobic surface area for air contact while preventing electrolyte leakage [12, 114]. The current collector, typically made of conductive metal mesh, such as Ni foam or stainless steel, is positioned between the active layer (which covers the current collector surface and interacts with the electrolyte) and GDL (which faces the open air) [115–117]. During the ORR process, the gaseous phase of oxygen is preferred due to its limited solubility and diffusivity in the electrolyte. Consequently, the air electrode’s high surface area provides a boundary between the gas (air), liquid (electrolyte), and solid (catalyst) [118]. Hence, air electrodes benefit from a 3D porous structure. Recently, innovative bifunctional catalysts have demonstrated enhanced performance in air cathodes, showing excellent efficiency, economic viability, and low pollution levels [119, 120]. However, the issue of catalyst and carbon substrate corrosion requires attention. Specifically, the reactive oxygen generated during OER can significantly damage and oxidize the catalyst and carbon material, primarily due to the larger surface area of carbon substrate [121].

The air electrode plays a crucial role in determining the overall performance of a battery as it facilitates the kinetics of oxygen reaction. The slow reaction kinetics of the air electrode during the ORR and OER contribute to the high polarization and poor electrode reversibility of ZABs. Thus, there has been significant interest in designing efficient bifunctional oxygen catalysts to accelerate the reaction kinetics and reduce charge/discharge overpotential, thereby improving battery performance. Spin regulation of catalytically active sites is the pioneering strategy in boosting oxygen reaction activity of catalyst [122]. Recently, Li et al*.* synthesized spin regulated heteroatom-doped amorphous transition metal sulfides (i.e., Mo-doped CoS) via a one-step hydrothermal process. The spin state of Co^2+^ was successfully modulated to the low-spin state, which optimized the adsorption free energy of various intermediates thereby enhancing the oxygen reduction reaction kinetics. The fabricated ZABs also delivered good cycle stability (over 100 h), a high discharge voltage (1.25 V under 0.5 A), and a superior overall mass-energy density (93 Wh kg^−1^), providing new insight into the design of efficient catalysts for oxygen electrocatalysis [123]. Various materials, including metal oxides, metal hydroxides, metal sulfides, carbon materials, and their composites, have been extensively explored as potential bifunctional oxygen electrocatalysts.

## Design of Air Catalysts

Bifunctional oxygen electrocatalysts with high activity and robust stability are essential for practical ZAB devices. In general, oxygen electrocatalysis takes place at the gas (O_2_)/solid (catalyst)/liquid (electrolyte) three-phase interface, and its intrinsic activity is closely related to the unsaturated coordination sites at the solid catalyst interface. Therefore, controlling the local electronic structure and surface/interface properties through unique design strategies is particularly important to optimize the adsorption/desorption behavior of intermediates, lower the energy barrier, and accelerate the kinetic process of ORR and OER reaction. In addition, the conductivity, number and intrinsic electrochemical activity of the active sites of ORR and OER catalysts are also important factors in achieving good performance of ZAB. Theoretically, the intermediate absorption energy barrier can be used to evaluate the intrinsic catalytic activity of an electrocatalyst. Several surface, near-surface, and interfacial engineering strategies have been explored to tune the electronic structure and binding energy of ZAB electrocatalysts. These strategies have deepened the understanding of the activity enhancement mechanism and provided important insight for the construction of high-efficiency bifunctional air cathode catalysts [16, 124]. In this section, various oxygen electrocatalyst engineering strategies including bifunctional oxygen electrocatalysts, perovskite oxides electrocatalysts, carbon-based electrocatalysts, and hybrid or mixed electrocatalysts are systematically described with typical examples of each design strategy which could provide in-depth understanding of the role of micro/nanostructure, surface, and electronic features of the air cathode in accelerating the kinetics of air electrocatalysts for high-speed ZAB.

### Bifunctional Oxygen Electrocatalysts

Despite the impressive electrocatalytic activity demonstrated by noble metal-based electrocatalysts like Ru, Ir, and Pt for the ORR and OER [125–127], the high manufacturing costs, limited lifespan, and inadequate bifunctional oxygen activity have hindered their use in rechargeable ZABs. As an alternative, there has been recent exploration of transition metal oxides, sulfides, phosphides, and carbon materials as highly efficient alternative materials [128–134]. Transition metal oxides (TMOs), especially cobalt oxides, have gained attention for their earth abundance, stable nature, and excellent bifunctional activity [32, 135, 136]. However, cobalt oxides still have limited electrocatalytic oxygen activity due to a scarcity of active sites and lower intrinsic activity of oxygen generation and dissociation [137]. To address this, porous nanostructures and the N doping of cobalt oxides have been proposed as effective approaches to increase active sites and enhance oxygen adsorption [138, 139]. Notably, the production of N-doped cobalt oxides necessitates high temperatures (> 600 °C) [136] or hazardous ammonia sources [140, 141], leading to challenges such as severe aggregation, structural breakdown, and environmental pollution problems [142].

**Fig. 6:**
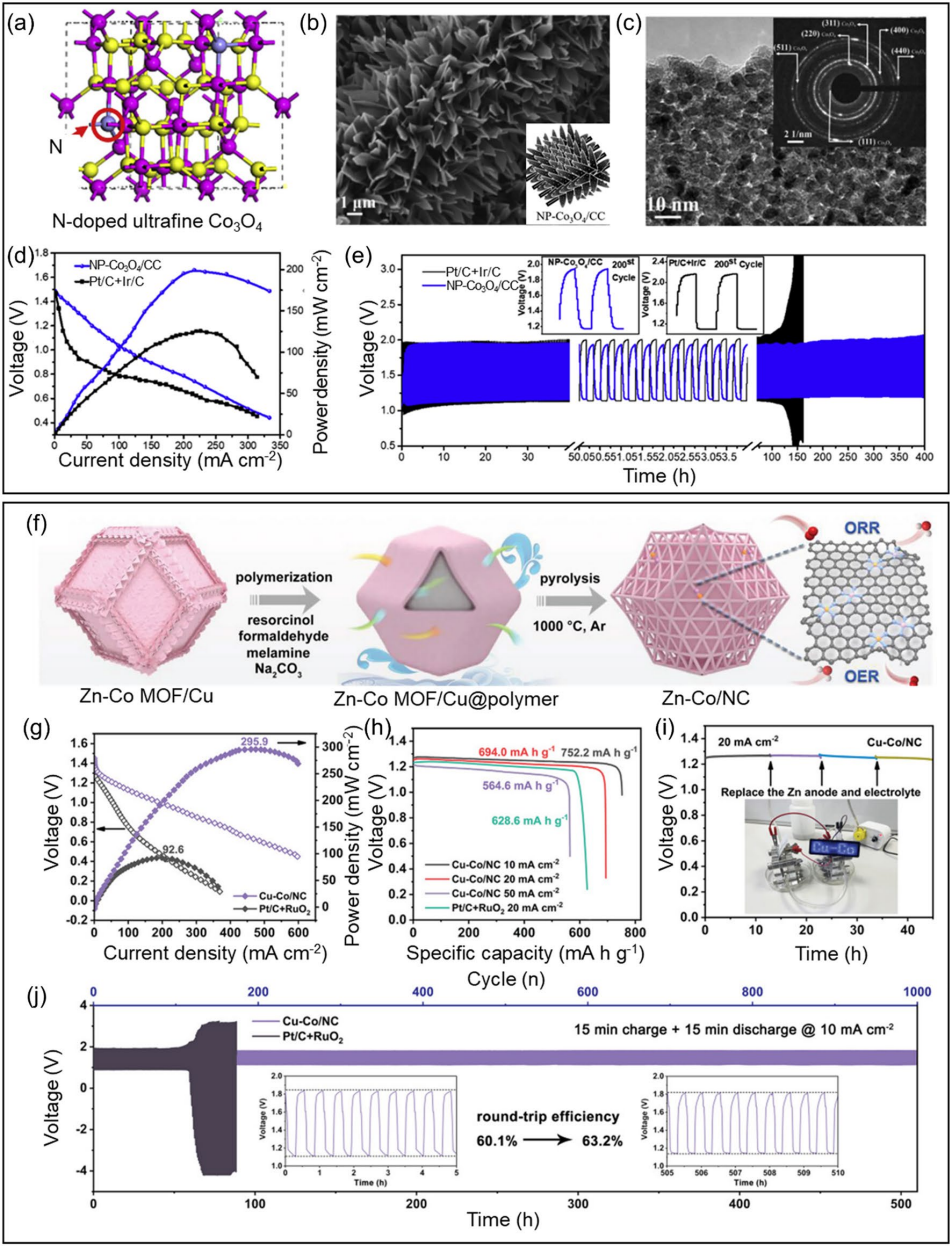
**a** Scheme showing the preparation of NP-Co_3_O_4_/CC and associated reaction mechanisms. **b** SEM micrographs for NP-Co_3_O_4_/CC. **c** HRTEM images for NP-Co_3_O_4_. **d** Battery voltage and power density and **e** Galvanostatic discharge–charge cycling curves at 5 ​mA ​cm.^−2^ of aqueous rechargeable ZABs with the NP-Co_3_O_4_/CC and Pt/C ​ + ​Ir/C catalyst as the air electrode, respectively. Produced with permission from [143]. **f** Synthetic scheme of Cu-Co/NC. **g** Discharge polarization curves and the corresponding power densities. **h** Specific capacities of zinc–air batteries at different discharge current densities. **i** Long-term durability of primary zinc–air battery with Cu-Co/NC catalyst. **j** Galvanostatic discharge/charge cycling curves (the inset shows the round-trip efficiency of zinc–air battery at first 10 cycles and last 10 cycles). Produced with permission from [144]

Wang et al. [143] synthesized porous nanoarrays consisting of N-doped cobalt oxide over carbon cloth (NP-Co_3_O_4_/CC) (Fig. 6a). The leaf-like vertically aligned nanoarrays on the carbon cloth substrate with the formation of abundant pores indicated on surface morphology of NP-Co_3_O_4_/CC (Fig. 6b). The diffraction rings presented in the SAED pattern were attributed to the lattice planes of Co_3_O_4_. Various facets of Co_3_O_4_ formed, which are indicated by planes (400), (311), (220), and (111) in the TEM images (Fig. 6c). Moreover, the ZIF-derived porous nanosheets of cobalt oxide resulted in NP-Co_3_O_4_ with a surface area of 173 m^2^ g^−1^, significantly greater than some previous works on porous cobalt oxides. The prepared NP-Co_3_O_4_/CC provided a highly effective cathode alternative, better than Pt/C + Ir/C air electrodes in terms of less overpotential, a higher power density (~ 200 mW cm^−2^), and a slight voltage drop after cycling for 400 h **(**Fig. 6d, e).

Wang et al. [144] proposed highly porous N-doped carbon matrix with copper–cobalt diatomic sites (Cu–Co/NC) for bifunctional (ORR/OER) oxygen electrocatalysts (Fig. 6f). The coexistence of CuN_4_ and CoN_4_ coordination boosted electrocatalytic activity. From the practical test of home-built ZAB setup, Cu–Co/NC delivered maximum peak power density of 295.9 mW cm^−2^ compared with Pt/C + RuO_2_ electrocatalyst for 92.6 mW cm^−2^ (Fig. 6g). The specific capacity and zinc utilization of Cu-Co/NC are 694 mAh g^−1^ and 85% at 20 mA cm^−2^, whereas 628.6 mAh g^−1^ and 77% for Pt/C + RuO_2_ at same conditions (Fig. 6h). There is no voltage drop during galvanostatic discharge at 20 mA cm^−2^ for 45 h and successfully operating LED device (Fig. 6i). Moreover, Cu–Co/NC demonstrates small voltage gap of 0.68 V over 510 h during 1000 charge–discharge cycles with negligible voltage decay (Fig. 6j).

Nanostructured manganese oxides (MnO_x_) have also been employed as active metal catalysts for the ORR and OER due to their abundance, multiple oxidation states (MnO, MnO_2_, and Mn_2_O_3_), large surface area, and higher electrocatalytic activity [145, 146]. Gorlin et al*.* [147] found that MnO_x_ is effective as a bifunctional catalyst, demonstrating electrode oxygen activity that is comparable with that of benchmark noble metal catalysts. Kim et al*.* [148] studied a Ni-doped Mn_2_O_3_ catalyst, showing a half-wave potential of ~ 0.801 V with a power density of 88.2 mW cm^−2^ for the prepared ZAB. Moreover, Li et al*.* [149] prepared Fe-doped hollow yolk-shelled Mn_3_O_4_ nanoboxes, resulting in a half-wave potential of 0.78 V for the ORR with a specific capacity of 740 mAh g^−1^. However, despite its potential as an effective catalyst for the ORR and OER, the practical application of MnO_x_ in long-life batteries is hindered by its low conductivity and instability [150].

Spinel oxides (AB_2_O_4_) have gained significant attention recently as bifunctional oxygen electrocatalysts due to their low toxicity, modifiable ion arrangement, and lower cost [151]. In particular, the presence of donor–acceptor adsorption sites can increase the catalytic activity of oxygen by facilitating the adsorption/desorption of reversible oxygen species in materials with mixed valences [152]. Doping AB_2_O_4_ with a third metal can also produce a more effective electronic structure, leading to stronger overall performance. For example, Liu et al*.* [153] deployed a solvothermal technique to produce a ZnCoMnO_4_/N-reduced graphene oxide (rGO) electrocatalyst. The Zn doping resulted in Co–N electronic interactions and consequently more favorable binding energies in ZnCoMnO_4_/N-rGO for O_2_ and H_2_O, which in turn produced outstanding ORR and OER performances.

**Fig. 7:**
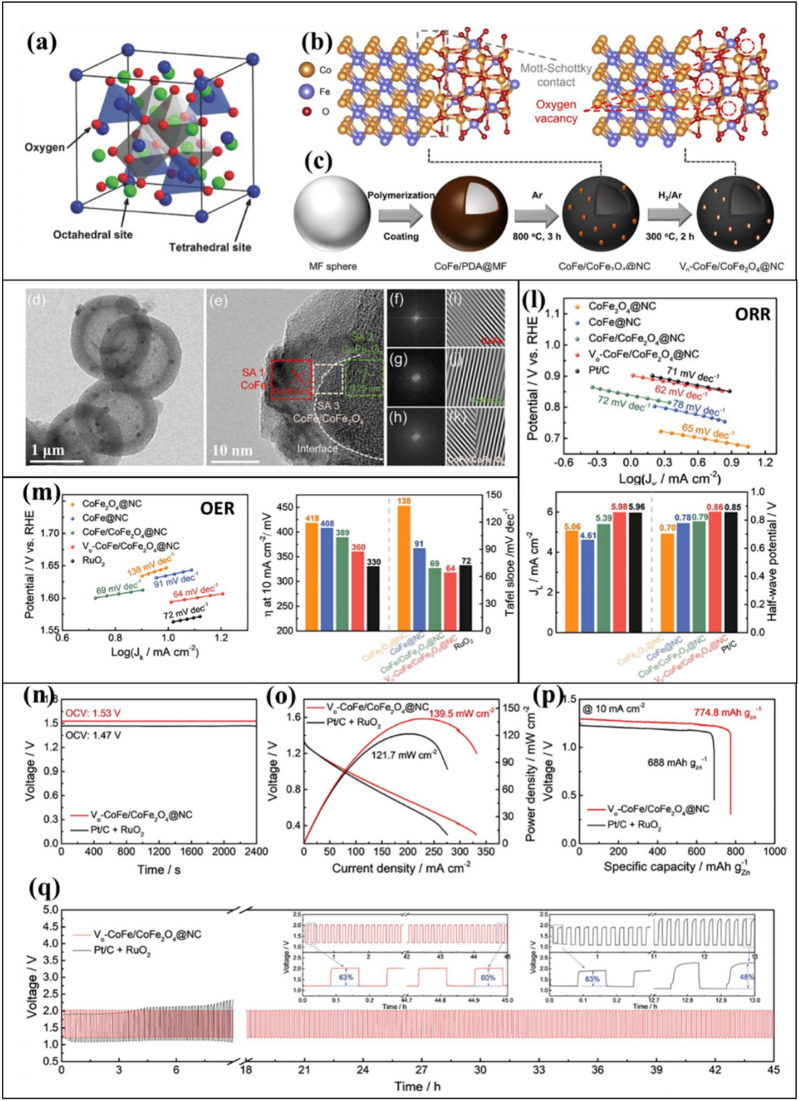
**a** Illustration of the spinel crystal structure. Reproduced with permission from [154]. **b, c** Crystallographic arrangement and step-by-step illustration of the synthesis of hollow-structured V_o_-CoFe/CoFe_2_O_4_@NC. **d** Low-resolution TEM image showing the hollow structure of V_o_-CoFe/CoFe_2_O_4_@NC. **e** HRTEM micrograph indicating the presence of CoFe, CoFe_2_O_4_, and CoFe/CoFe_2_O_4_ and the location of the heterointerface between CoFe and CoFe_2_O_4_. **f–h** Corresponding fast Fourier transform (FFT) and **i–k** inversed FFT micrographs. **l** ORR performance in terms of the Tafel plot (top) and the current density and half-wave potential (bottom) for the prepared catalysts in 0.1 M KOH with O_2_ saturation. **m** OER performance in terms of the Tafel plot (left), the OER overpotential (right) required to achieve a current of 10 mA cm^−2^, and the Tafel slopes for the prepared catalysts in 0.1 M KOH with N_2_ saturation. **n** Comparative analysis of the open-circuit voltage (OCV) measured for V_o_-CoFe/CoFe_2_O_4_@NC and Pt/C + RuO_2_. **o** Polarization curve and plots of the power density. **p** Galvanostatic full-discharge test at a fixed current density of 10 mA cm.^−2^. **q** Charge/discharge cyclic performance for ZABs using V_o_-CoFe/CoFe_2_O_4_@NC (red line) and Pt/C + RuO_2_ (black line) as the air cathode. Reproduced with permission from [158]

Spinels have the potential to catalyze both the ORR and OER (Fig. 7a) due to the presence of both tetrahedral and octahedral sites inside the structure [154]. As such, tuning the oxidation states or oxygen vacancies is possible via the rational partial substitution of tetrahedral or octahedral sites within the crystal lattice. Recently, researchers have reported the development of nano-sized NiCo_2_O_4_ and its nanocomposites, particularly those containing N-doped carbon nanostructures [155–157]. The abundance of edge active sites in 1D morphologies such as carbon nanotubes (CNTs) and carbon nanofibers improves electrochemical metrics and leads to longer stability when combined with NiCo_2_O_4_. Similar outstanding results have been achieved when CNTs are combined with spinels derived from other transition metals (*e.g.,* Co, Fe, Mn, and Zn). Zhao et al*.* [126] demonstrated a facile strategy based on oxidative thermal treatment. Using this strategy, the residual Mn and Co oxide NPs embedded within the N-doped CNTs were transformed into spinel Mn-Co oxide NPs partially incorporated in the N-doped CNTs. Due to the close proximity of the spinel Mn-Co oxide and the graphitic walls of the CNTs, the resulting catalyst exhibited a strong bifunctional ORR and OER performance.

Most recently, Go et al*.* [158] reported oxygen-vacancy-rich CoFe/CoFe_2_O_4_ incorporated in N-doped hollow carbon spheres (Fig. 7b, c). Figure 7d presents a TEM image of the hollow structure of the carbon spheres, while Fig. 7e displays a high-resolution TEM (HRTEM) image of the prepared catalysts (CoFe, CoFe_2_O_4_, and the composite CoFe/CoFe_2_O_4_) and the heterointerface between CoFe and CoFe_2_O_4_. Figure 7f–h present fast Fourier transform (FFT) micrographs showing the mixed crystal structure of the composite. Additionally, high-resolution inverse FFT (IFFT) micrographs (Fig. 7i–k) were used to determine the lattice spacing for the prepared catalysts, with the results revealing (1 1 0) and (2 2 0) planes corresponding to the CoFe alloy and CoFe_2_O_4_. The coexistence of various crystal structures and a heterointerface between the CoFe and CoFe_2_O_4_ was also observed. Figure 7l, m presents the results for the ORR and OER performance of the fabricated ZAB. V_o_-CoFe/CoFe_2_O_4_@NC demonstrated excellent ORR performance with a half-wave potential of 0.855 V and a Tafel slope of 62 mV dec^−1^, which was a significant improvement over Pt/C. The OER performance of the prepared spheres (overpotential of 360 mV; Tafel slope of 64 mV dec^−1^) was also higher than that of the state-of-the-art RuO_2_ which had an overpotential of 330 mV and a Tafel slope of 72 mV dec^−1^. A high OCV of about 1.53 V, a high current density of 139.5 mW cm^−2^, an excellent specific capacity of 774.8 mAh g_Zn_^−1^, and remarkable stability for up to 45 h were observed when the V_o_-CoFe/CoFe_2_O_4_@NC composite was used as the air cathode in a ZAB. The excellent ORR, OER, and ZAB performance was due to the high number of oxygen vacancies in CoFe_2_O_4_ and the strongly coupled heterointerface between the CoFe_2_O_4_ and CoFe alloy.

### Perovskite Oxides Electrocatalysts in Alkaline Electrolytes

Perovskite oxides (ABO_3_) containing alkali or rare-earth metals at the A-site and a transition metal at the B-site have gained great attention as cathode electrocatalysts [159–162]. Perovskite oxides offer a diverse range of structures, oxygen levels, and electrocatalytic features that are tunable with the partial replacement of A- or B-site cations [163, 164]. A number of strategies such as cation doping, nanostructuring, surface optimization, and the use of composites can improve ORR/OER performance [165, 166]. Zhu et al*.* [167] proposed a novel SrNb_0.1_Co_0.7_Fe_0.2_O_3−*δ*_ (SNCF) perovskite and studied its stability and activity in an alkaline solution, finding that this material has the potential to act as an electrocatalyst in the OER process. The author observed that the addition of niobium to the A-site of SrCo_0.7_Fe_0.2_O_3_ resulted in improved OER performance. Bu et al*.* [165] reported a novel approach for the fabrication of cation-ordered perovskites as efficient bifunctional catalysts for ZABs. The author optimized the Fe content in PrBa_0.5_Sr_0.5_Co_2–*x*_Fe_*x*_O_5+δ_ (where *x* = 0 − 2) and observed an OER performance that was almost nine times higher than that of the noble metal oxide IrO_2_.

**Fig. 8:**
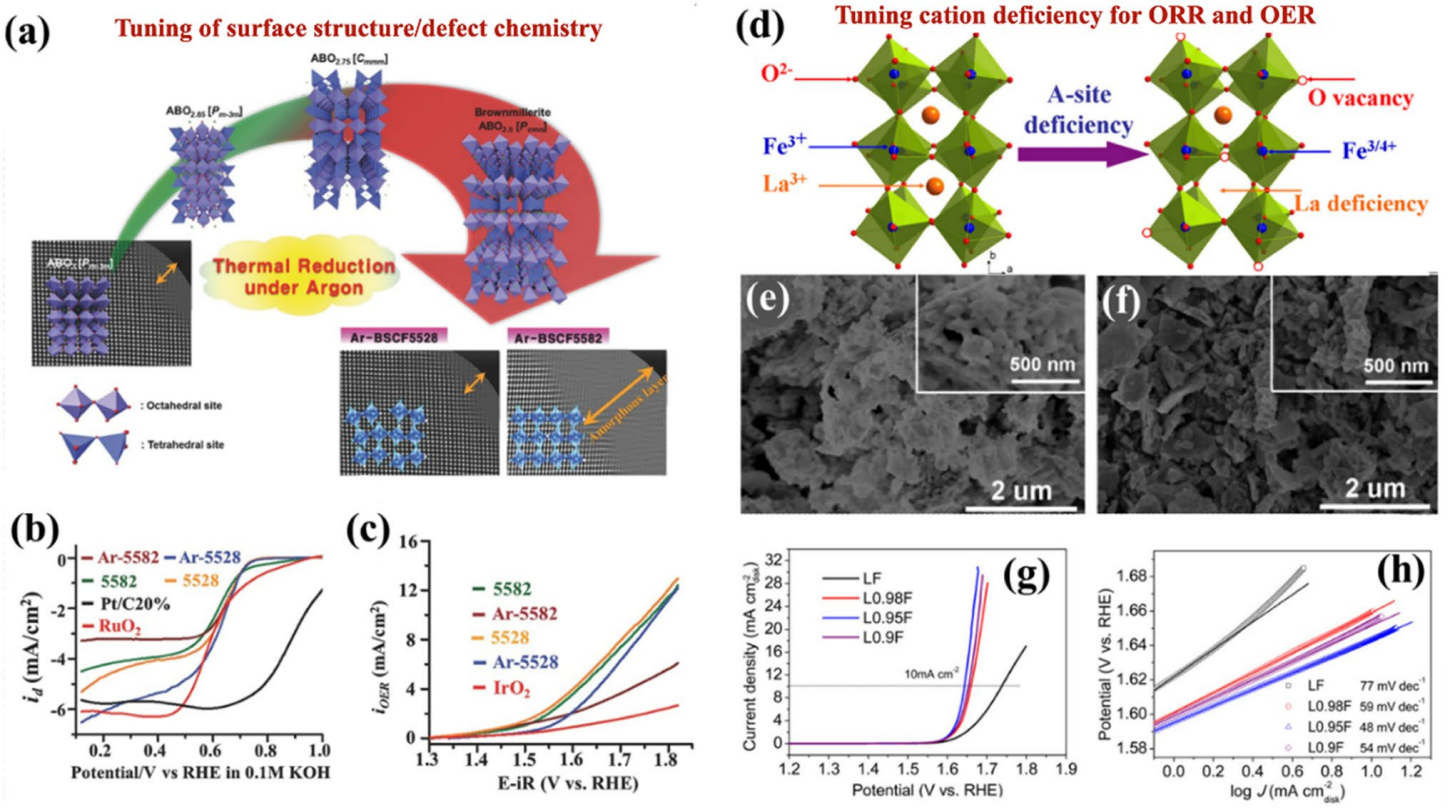
**a** Structural change in response to heat treatment (950 °C/24 h) in an argon (Ar) environment. **b** ORR performance of modified perovskites and standard catalysts. **c** Linear plots for OER activity. Reproduced with permission from [156]. **d** A-site cationic deficiency strategy showing the crystal structures for the original LF and the oxygen vacancies in the modified La_1–*x*_FeO_3-δ_ perovskites. **e, f** SEM micrographs for the original LF (left) and optimal perovskite L_0.95_F (right). **g** Linear voltammograms used to determine the OER performance of the original and modified perovskite catalysts and **h** corresponding Tafel plots. Reproduced with permission from [168]

Defect engineering is also a useful technique for a range of electrocatalytic processes. Defects in metal oxides can significantly alter the material's band structure, spin state, and charge transport. The most observed defect in perovskite oxides is oxygen vacancies, the presence of which increases electrocatalytic performance. Two common paths to inducing oxygen vacancies in perovskite oxide electrocatalysts are treating them with reductive gases/agents or producing more/fewer A-sites. Jung et al*.* [159] reported the heat treatment (950 °C) of Ba_0.5_Sr_0.5_Co_*x*_Fe_1-*x*_O_3-δ_ (where *x* = 0.2 or 0.8; BSCF5582 and BSCF5528) in an argon (Ar) environment and investigated the resulting structural surface, defect chemistry, and electrocatalytic performance (Fig. 8a–c). After treating the original perovskite (*P*_*m-*3* m*_) in a reduction environment under Ar, N_2_, or a vacuum, a modified perovskite structure with the oxygen-deficient brownmillerite phase *P*_*cmn*_ was obtained. This environment induced oxygen vacancies in the perovskite structure, leading to square planer or local tetrahedral defect sites (Fig. 8a). Samples BSCF5582 and BSCF5528 were in an Ar atmosphere at 950 °C. Interestingly, BSCF5582 had an amorphous layer with a thickness that was 10 times larger than the original structure, resulting in lower electrocatalytic performance. However, the amorphous layer was slightly less thick for sample BSCF5528 but with a larger concentration of oxygen vacancies, leading to stronger ORR performance. Thus, tuning the surface structure and/or defect chemistry is a viable approach to achieving high electrocatalytic performance. Using disk electrodes, the ORR performance of Ar-BSCF5582 and Ar-BSCF5528 perovskites was compared with that of RuO_2_ and Pt/C (Fig. 8b). The calculated onset potential and limiting current density demonstrated that Ar-BSCF5528 was the highest-performing candidate. The OER activity of both samples was also evaluated with and without Ar (Fig. 8c). The results indicated that heat treatment had a different impact on the structure and defect chemistry of BSCF5582 and BSCF5528. The OER activity for Ar-BSCF5582 was significantly lower due to the thickness of the amorphous layer, while Ar-BSCF5528 did not exhibit a significant change in the amorphous layer thickness following the heat treatment.

Zhu et al*.* [168] reported enhanced electrocatalytic activity for perovskite oxides La_1–*x*_FeO_3-δ_ (where *x* = 0.02, 0.05, 0.1) in an alkaline solution, employing a tunable cationic deficiency method. Figure 8d presents the crystal structure tuning used to produce oxygen vacancies via the A-site deficiency strategy, while Fig. 8e, f displays SEM micrographs at different resolutions (2 µm and 500 nm) of the original LaFeO_3_ (LF) and modified La_0.95_FeO_3-δ_ (L_0.95_F) perovskites. The SEM micrographs show that the A-site cation-deficient perovskite L_0.95_F (Fig. 8f) had a significantly lower particle size and the largest surface area, leading to additional active sites and enhanced ORR and OER activity. As illustrated in Fig. 8g, the OER activity was measured using linear sweep voltammetry (LSV). It is interesting to note that L_0.95_F attained a current density of 10 mA cm^−2^ at a lower onset potential of 1.64 V, whereas the original LF sample did not achieve this current density until it reached an onset potential of 1.74 V. This was accredited to the more abundant active sites in L_0.95_F compared with LF. Additionally, the Tafel slopes for LF and cation-deficient La_1–*x*_FeO_3-δ_ are presented in Fig. 8h. Significantly smaller Tafel slopes were observed for La_1–*x*_FeO_3-δ_, which were attributed to the rapid OER rate and the improved charge transfer capability of the modified LF. Thus, tunable cationic deficiencies represent a useful strategy for producing perovskite oxide-based bifunctional materials with excellent electrocatalytic performance.

### Carbon-Based Electrocatalysts for ZABs

Precious-metal-based electrocatalysts including Pt and its alloys are highly effective for the ORR but display poor OER activity due to the formation of insulating Pt oxides with low electrical conductivity [169]. Conversely, metal oxides such as RuO_2_ and IrO_2_ exhibit excellent OER performance but are less efficient for the ORR [138]. However, the use of these novel metal oxides in Zn–air batteries (ZABs) is restricted due to their limited availability, high cost, and instability. Consequently, substantial efforts have been dedicated to developing alternative materials with superior bifunctional OER and ORR performance [170–172]. These materials include TMOs, hydroxides, sulfides, metal-free carbon, and carbon-containing transition metals. Carbon-based materials, such as graphene/rGO, CNTs, and their hybrids, have demonstrated rapid electron transfer and impressive ORR and OER performances. Additionally, their structural properties can be tailored using a variety of strategies such as heteroatom doping (*e.g.,* N, B, O, S, and P) and defect engineering, which leads to the preferential generation of OH^−^ through a four-electron pathway [173, 174].

The presence of more electronegative heteroatoms (as compared to carbon atoms) may be responsible for the enhanced ORR of heteroatom-decorated carbons because this creates electron deficiencies or structural disorder in neighboring carbon atoms, leading to facile oxygen adsorption on the carbon surface [39, 175, 176]. With N doping, for example, three distinct active sites can be formed when N is introduced to C: graphitic N (quaternary N), pyrrolic N, and pyridinic N [176]. Graphitic N provides electrons to the p-conjugated system, which can increase the nucleophilic nature of the surrounding carbon rings and an increase in O_2_ adsorption on the carbon surface. Pyridinic N, on the other hand, has the ability to attract electrons from neighboring carbons and expedite the adsorption of H_2_O oxidation intermediates, which can lead to greater OER activity. Liu et al*.* [177] experimentally verified bifunctional active sites in N-doped graphene nanoribbons and reported that quaternary-N and pyridinic-N sites were responsible for the ORR and OER performance, respectively. The bifunctionality of N-doped graphene nanoribbons induces a synergistic effect, enhancing catalytic activities and stability through the electron-donating and electron-withdrawing nature of quaternary and pyridinic-N sites, which are favorable for ORR and OER, respectively. The assembled ZAB demonstrated an excellent power density of 65 mW cm^−2^ with remarkable cyclic stability over 30 h. Collectively, these studies have illustrated the importance of heteroatom-doped carbon for use in metal-free carbon bifunctional electrocatalysts.

**Fig. 9:**
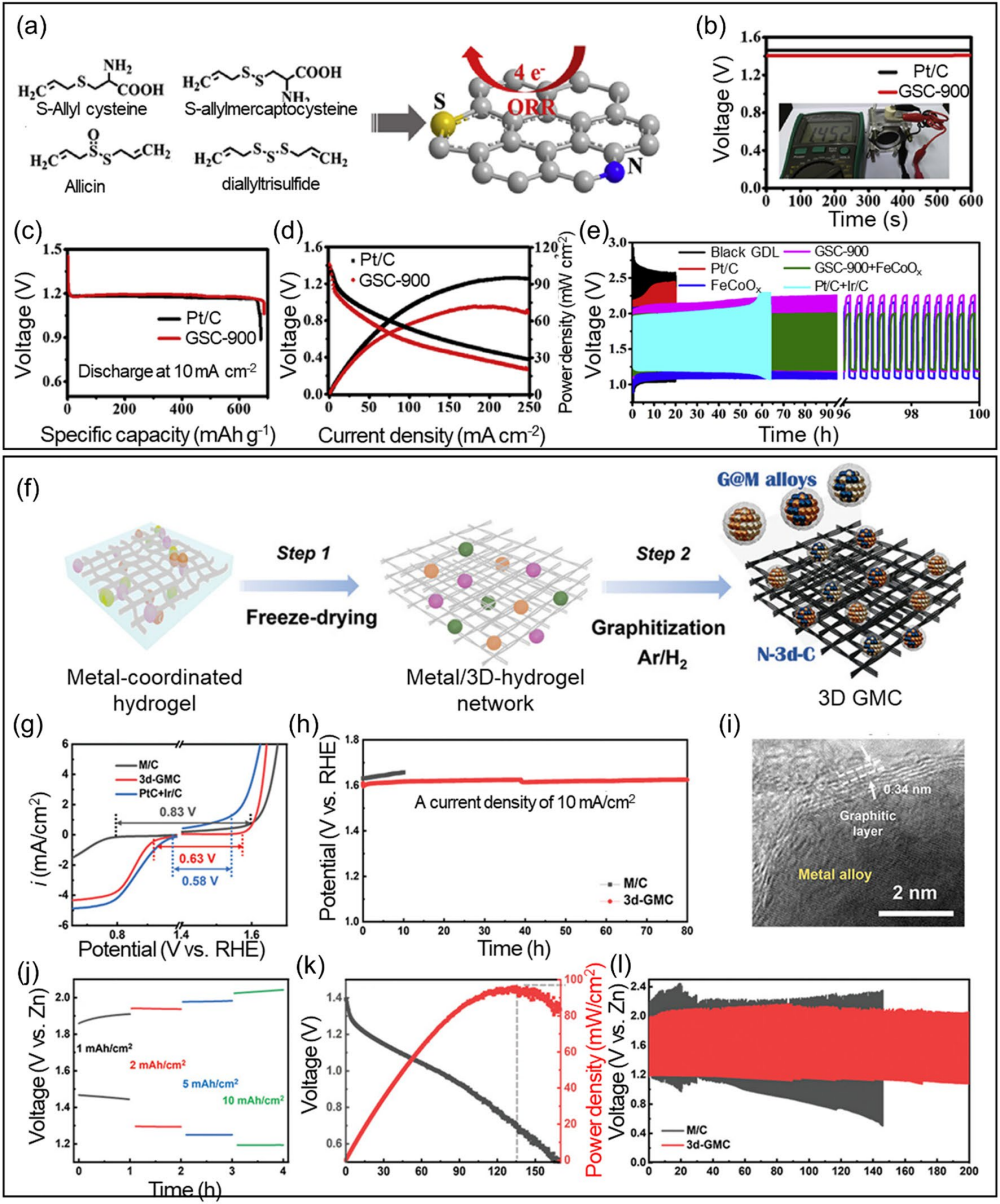
**a** Preparation process for heteroatom (N and S) co-doped porous carbon derived from garlic stems. **b** OCV for a primary ZAB containing GSC-900 and Pt/C as air cathodes. The inset presents the voltage measured using a multimeter. **c** Galvanostatic discharge curves for the primary ZABs to assess their specific capacity. **d** *V-J* measurements and related power density for ZABs using GSC-900 and Pt/C as air cathodes. **e** Galvanostatic cycles for different electrocatalysts and their physical mixtures when used as the air cathode in a rechargeable ZAB. **f** Schematic illustration of the simple preparation process of 3d-GMC from the metal-coordinated hydrogel. **g** Bifunctionality of M/C (black), 3d-GMC (red), and Pt/C + Ir/C (blue): the bifunctionality value (i.e., onset potential differences (ΔE) between ORR and OER) for each catalyst is provided in figs. **h** Durability test of M/C (black) and 3d-GMC (red) at a current density of 10 mA cm^−2^. The electrocatalytic test was performed using 0.1 m KOH as an electrolyte. **i** TEM images for 3d-GMC after OER test. Full cell performance of 3d-GMC for Zn–air battery. **j** Rate capability, **k** polarization tests demonstrating power density, and **l** cyclability for a Zn–air battery assembled using a 3d-GMC cathode (red) and commercial Zn foil anode compared with the Zn–air battery using M/C as the cathode (black). The rate capability test was conducted in this order: OCV, 1, 2, 5, 10 mA cm.^−2^. Reproduced with permission from [179]

By controlling the electronic structure and surface polarity, dual-atom doping can also enhance the electrocatalytic performance of carbon materials. Ma et al*.* [178] synthesized N- and S-doped porous carbon using the self-activation strategy on garlic stems and demonstrated improved ORR electrocatalytic activity and overall ZAB performance. Figure 9a presents the complete preparation process for heteroatom-doped porous carbon. The compounds containing N and S are present in the garlic stems, and these interact with the graphitic rings of the carbon. The high conductivity from graphitic structures and electron acceptor/donor characteristics of N/S heteroatoms promoted catalytic activity for improving ZAB performance. The doped carbon was used as an air cathode in a primary ZAB and the resulting electrocatalytic activity was monitored. The OCV for the ZABs with heteroatom-doped carbon (GSC-900) and standard Pt/C was estimated to be 1.46 V and 1.41 V, respectively (Fig. 9b). Additionally, the calculated specific capacity was around 685 mAh g^−1^ and 674 mAh g^−1^ for GSC-900 and Pt/C, respectively. Moreover, the power density was around ~ 95 mW cm^−2^ and ~ 72 mW cm^−2^ for GSC-900 and Pt/C, respectively (Fig. 9c). The galvanostatic pulse cycles were analyzed for the electrocatalysts, and their physical mixtures were used as the air cathode in a rechargeable ZAB **(**Fig. 9d), with GSC-900 + FeCoOx outperforming its competitors. This high performance was ascribed to the collaborative role of N- and S-doped elements in the carbon and metal species, which promoted the ORR and OER. Jang et al. [179] reported 3D-metal (Co, Fe, and Ni alloys)-coordinated hydrogel in situ-grown graphene on N-doped carbon supports (3d-GMC) (Fig. 9e). The bifunctionality values of 3d-GMC are 0.63 V, indicating that encapsulating 3D graphene onto transition metal alloys enhanced the bifunctionality (Fig. 9f). The 3d-GMC represents superior durability with no potential drop for 83 h at an operating potential of 1.62 V at 10 mA cm^−2^ and excellent chemical stability with no structural destruction after operation (Fig. 9g-h). The catalytic activity under high-current density operating represents rate capability of 85% retention (1.1 and 1.3 V at 10 and 1 mA cm^−2^, respectively) and maximum power density of 100 mW cm^−2^. The successive charge/discharge cycles tested during 200 h operated at 5 mA cm^−2^ for 5 min.

Another effective strategy to enhance bifunctional oxygen electrocatalysts for use in ZABs is N and P co-doping to produce porous carbon. Zhang et al*.* [18] prepared mesoporous carbon via N and P co-doping that had a large specific surface area of ∼1663 m^2^ g^−1^ Introducing co-doped N/P heteroatoms effectively controlled the electronic characteristics and surface polarities, resulting in improved ORR and OER activities. The outcomes indicated that air electrodes comprising N and P-doped porous carbon exhibited exceptional performance in both primary and rechargeable ZABs. An OCV of 1.48 V, a specific capacity of 735 mAh g_Zn_^−1^, and a power density of 55 mW cm^−2^ with stable operation for over 240 h were observed for the primary ZAB, while the rechargeable ZAB demonstrated excellent stability over 180 cycles at 2 mA cm^−2^. Density functional theory simulations suggested that co-doping of N/P with the highly porous framework of the prepared carbon material was critical to its bifunctional activity for the ORR/OER processes. Specifically, coupled graphitic structures with N/P co-doping exhibited the lowest overpotentials for both ORR and OER, while isolated N or P-doped graphitic structures displayed higher overpotentials.

The ternary heteroatom doping of porous carbon has also been reported to achieve higher electrocatalytic activity than single-doped carbon materials due to the synergistic effect of the heteroatom elements [180–182]. Wang et al*.* [183] reported ternary heteroatom (N, B, and F) doping in carbon fibers using electrospinning and annealing. The prepared heteroatom-doped carbon fibers demonstrated superior ORR activity and specific capacity of 555 mAh g_Zn_^−1^ with remarkable stability and reversibility after continuous cycling for 130 h at 10 mA cm^−2^. Razmjooei et al*.* [184] prepared ternary N, S, P-doped rGO using thiourea as the N/S dopant and triphenylphosphine as the P dopant. The prepared ternary heteroatom-doped rGO demonstrated outstanding ORR activity, nearly twofold higher than the dual N/S-doped rGO, and almost five times higher than P-doped rGO. This excellent ORR performance is accredited to the synergistic role of ternary heteroatom elements (N, S, and P) which not only create additional active sites but also increase the graphitic order and the surface area due to the greater mesopore volume.

**Fig. 10:**
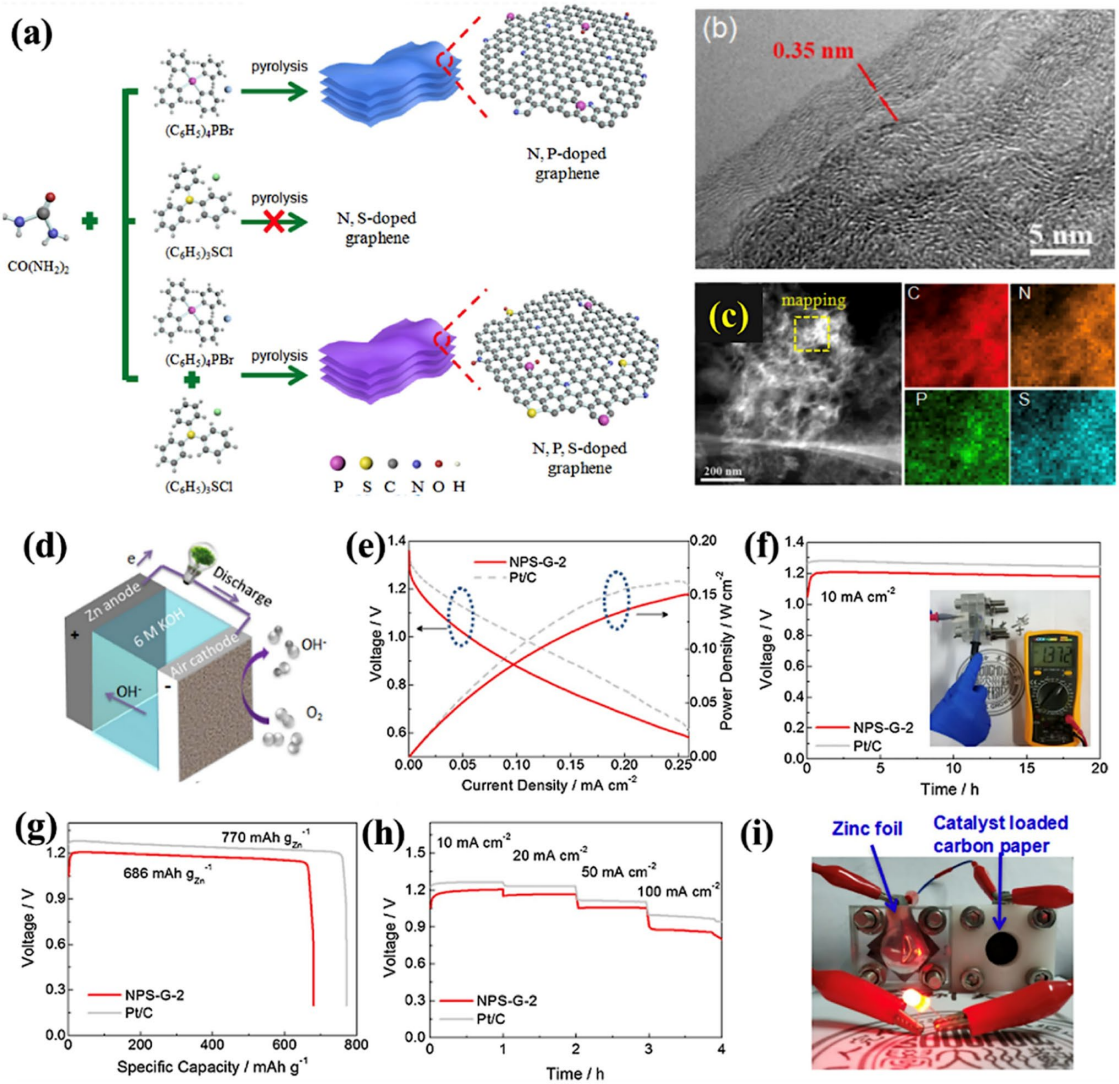
**a** Process for the fabrication of N,P-doped graphene, N,S-doped graphene and N,S,P-doped graphene (NSP-G). **b** HRTEM micrograph of NSP-G. **c** STEM micrograph of the prepared NPS-G sample and elemental mapping to determine the heteroatom content (N, P, and S). **d** 3D diagram of the ZAB. **e** Power density calculations and polarization curves for ZABs using NPS-G-2 and commercial Pt/C (with 20 wt.%) as the air cathode. **f** Galvanostatic discharge curves at 10 mA cm.^−2^ for the ZABs with NPS-G-2 and Pt/C as the air cathodes, showing an OCV of 1.372 V for NPS-G-2. **g** Specific capacity of the ZABs with NPS-G-2 and Pt/C as the ORR catalyst. **h** Discharge profiles for different current densities for the ZABs with NPS-G-2 and Pt/C as the air catalyst. **i** Photographic image showing illumination from a green LED powered by two liquid ZABs connected in series with NPS-G-2 as the air cathode. Reproduced with permission from [185]

Zheng et al*.* [185] reported a facile one-step pyrolysis strategy for the production of N-, S-, and P-doped graphene-like carbon using onium salts as precursors. Figure 10a presents a schematic illustration of the synthesis of N/P/S-doped graphene The higher electronegativity of N heteroatoms (3.04) compared to C (2.55) leads to the creation of charged carbon sites (C +), favorable for O_2_ adsorption and thus enhancing ORR activity. Moreover, the electron-donating nature of N, acting as an n-type dopant, augments electric conductivity and shifts the Fermi level closer to the conduction band. In contrast, P heteroatoms exhibit even greater electron-donating capacity than N due to their larger atomic radius (70 pm for N and 110 pm for P). The pronounced atomic radius of P induces significant distortion in graphitic structures and generates open edge sites, thereby further enhancing ORR activity. Additionally, the introduction of S dopants induces a high spin density, further promoting ORR activity. Surface morphological analysis using TEM and EDX (Fig. 10b, c) revealed that the heteroatom-doped material consisted of wrinkled nanosheets with an interlayer distance of ca. 0.35 nm, which was comparable to graphene (0.34 nm). EDX analysis also confirms the presence of N, S, and P in the prepared graphene-like carbon structure. After the successful synthesis of the heteroatom-doped graphene, the electrocatalytic performance of the resulting material (NPS-G-2) was tested using a primary ZAB in which electrocatalyst-containing carbon fiber acted as the air cathode (Fig. 10d). The power density and polarization curves were determined, and a peak power density of about 0.151 W cm^−2^ and an OCV of 1.372 V were achieved (Fig. 10e, f), representing remarkable electrocatalytic performance. The specific capacity was measured to be ~ 686 mAh g_Zn_^−1^ at 10 mA cm^−2^ (Fig. 10g), while the galvanostatic discharge voltage decreased with an increase in the current density (Fig. 10h). A green LED (2.4 V) was also powered with two liquid ZABs with an NPS-G-2 air cathode connected in series (Fig. 10i). The LED demonstrated remarkable operational stability without any degradation in the brightness of the light over 12 h.

### Hybrid or Mixed Electrocatalysts for ZABs

Efforts to design economical non-noble metal-based electrocatalysts, such as 3D transition metals [186] and nanocarbons, or their hybrid/composites, have been prompted by the high cost and scarcity of the noble metals currently used in commercially available catalysts (such as the Pt-based catalysts used for the HER and ORR and Ru/Ir-based catalysts used for the OER) [187, 188]. Because of their high catalytic performance, particularly for the HER and OER, transition metal phosphides (TMPs) (where TM = Fe, Mn, Co, Ni, Cu, and W) have maintained consistent research interest over recent years [189, 190]. However, improvements in electrocatalytic performance are hampered by their low surface area, insufficient electronic conductivity, and poor NP dispersion [191, 192]. To enhance the electrocatalytic performance of the prepared electrocatalyst, carbon is used to hold the TMP NPs, producing a hybrid TMP/C structure. Additionally, metal–organic frameworks (MOFs), which have a high SSA and tunable porous structures, are considered an ideal precursor for hybrid TMP/C via carbonization at elevated temperatures [192, 193]. Liu et al*.* [194] demonstrated a trifunctional electrocatalyst in which Co_2_P was implanted in heteroatom-doped (Co, P, and N) carbon (Co_2_P/CoNPC) using zeolitic imidazolate frameworks as a precursor. The synergistic effects of the heteroatom-doped carbon-based substrate and Co_2_P supported high electrocatalytic OER, HER, and ORR activity similar to that of commercially available Pt/C or RuO_2_ catalysts.

**Fig. 11:**
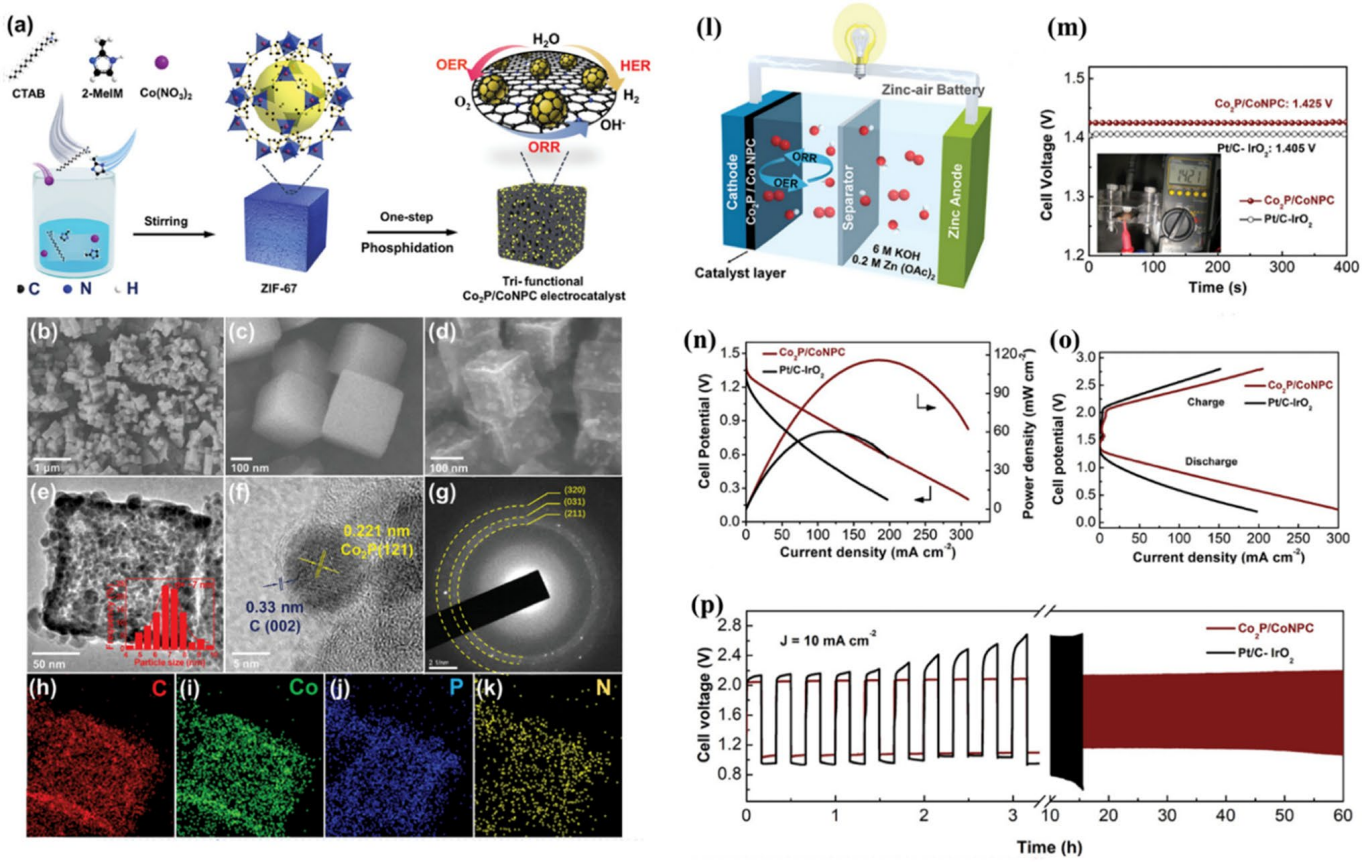
**a** Preparation process for Co_2_P/CoNPC. **b, c** SEM images for ZIF-67. **d** SEM image for Co_2_P/CoNPC. **e** HRTEM image for Co_2_P/CoNPC, with the inset showing the particle size distribution for Co_2_P NPs. **f** HRTEM image showing the planes related to Co_2_P NPs and the carbon framework. **g** SAED pattern. **h–k** Elemental mapping of Co_2_P/CoNPC. **l** Co_2_P/CoNPC used as the air cathode in a ZAB. **m** OCV plot with the inset showing the multimeter setup for the calculation of the voltage. **n** Plot for the power density. **o** Charge/discharge polarization data. **p** Cyclic stability performance for the assembled ZAB. Reproduced with permission from [194]

The preparation steps for Co_2_P/CoNPC are illustrated in Fig. 11a. SEM and TEM micrographs were acquired to assess the surface and morphology of the samples (Fig. 11b–f). These analyses clearly indicated that the prepared Co_2_P/CoNPC had an inherent ZIF-67 morphology, while the Co_2_P NPs were evenly dispersed within the carbon framework. The size of the smallest NPs was as low as 7 nm, which is why it was anticipated to offer additional active sites and achieve excellent electrocatalytic activity. Furthermore, Fig. 11f presents an HRTEM image that shows a lattice spacing of 0.221 nm, which was ascribed to the (1 2 1) plane of Co_2_P; a lattice spacing of 0.33 nm was also observed and ascribed to the carbon (0 0 2) plane, suggesting the presence of a carbon layer on the Co2P NPs. The selected area diffraction (SAED) measurements (Fig. 11g) also confirmed the presence of a carbon framework and Co_2_P crystals. To further visualize the heteroatom elements, elemental mapping micrographs were obtained (Fig. 11h–k). To assess the performance of the proposed trifunctional catalyst, a rechargeable ZAB was assembled with a Co_2_P/CoNPC-based air cathode (Fig. 11l). The ZAB produced a high OCV of 1.425 V (Fig. 11m), a peak power density of 116 mW cm^−2^ (Fig. 11n), a low charge–discharge voltage gap of around 1.13 V at 50 mA cm^−2^ (Fig. 11o), and negligible potential loss during the charge–discharge tests after 60 h (Fig. 11p). The authors ascribed this high electrocatalytic performance to the synergistic effect of Co_2_P and the heteroatom-decorated carbon.

Shi et al*.* [195] recently demonstrated a trifunctional electrocatalyst composed of FeCo NPs surrounded by graphitic carbon, Co_2_P NPs, and a N,P-doped carbon fiber framework. The synergistic effect of Co_2_P and FeCo NPs was responsible for enhancing the ORR, HER, and OER activity. Xia et al*.* [196] also demonstrated a self-standing Co/nanocarbon membrane fabricated using a facile electrospinning technique. This membrane was employed as a bifunctional air electrode in a ZAB and achieved a high power density of 304 mW cm^−2^ and a lifetime of 1500 h at 5 mA cm^−2^. This high performance was due to the self-standing membrane structure, which provided abundant Co–N–C active species inside the hierarchical electrode. Despite their promising performance, MOF-based strategies typically involve complex multistep processes that include the carbonization/oxidation of MOFs followed by phosphidation [197, 198]. In addition, MOF-based electrocatalysts suffer from particle agglomeration, degraded electrocatalytic activity, and low mechanical stability thus, limiting their performance when used in ZABs [199–201]. To resolve these issues, other advanced strategies are required, such as continuous oxygen electrocatalysts and self-standing air electrodes. Table 3 summarizes recently reported bifunctional catalysts with their performance in ZABs.

**Table 3:** Recent progress of bifunctional catalysts with their performance in ZABs

Type	Electrolyte	Air electrode catalyst layer	Zn electrode	Open circuit voltage (V)	Discharge/charge voltage gap (V)	Peak power density (mW cm^−2^)	Specific capacity (mAh g^−1^)	Cyclic performance	References
A	6 M KOH + 0.2 M Zn(CH_3_COO)_2_∙2H_2_O	N-CoS_2_ YSSs	Polished Zn foil	1.41	0.85 @ 10 mA cm^−2^	81	744	More than 165 h @ 10 mA cm^−2^	[202]
A	6.0 M KOH + 0.2 M Zn acetate	Porous Ni/NiO nanosheets	Zn plate	1.47	0.83 @ 2 mA cm^−2^	225	853	240 cycles, 120 h @ 2 mA cm^−2^	[203]
A	poly(vinyl alcohol) (PVA) gel film	Spinel CoIn_2_Se_4_ nanosheets	Zn plate	1.37	0.71 @ 10 mA cm^−2^	107	733	400 cycles @ 10 mA cm^−2^	[204]
A	6 M KOH + 0.2 M Zn acetate	MnO_2_-IL_0.5_	Polished Zn plates	1.51	0.86 @ 10 mA cm^−2^	166	762	40 h @ 10 mA cm^−2^	[205]
A	KOH + Zn(Ac)_2_	np-AlFeCoNiCr	Zn foil	1.55	0.76 @ 2 mA cm^−2^	125	800	120 h @ 20 mA cm^−2^	[206]
A,B	6 M KOH + 0.2 M Zn(OAc)_2_	Co_3_O_4_@LaCoO_3_	Zn plate	1.46	–	140	785	555 cycles, 185 h @ 2 mA cm^−2^	[207]
A,B	6 M KOH + 0.2 M Zn(OAc)_2_	LaMnO_3_	Zn foil	–	0.82 @ 10 mA cm^−2^	170	725	100 cycles @ 5 mA cm^−2^	[208]
A,B	6 M KOH + 0.2 M Zn(OAc)_2_	LaNi_0.85_Mg_0.15_O_3_	Polished Zn foil	1.35	0.92 @ 10 mA cm^−2^	45	810	220 cycles, 110 h @ 10 mA cm^−2^	[209]
A,B	6 M KOH + 0.2 M Zn(OAc)_2_	Ba_0.5_Sr_0.5_Co_0.8_Fe_0.2_O_3-δ_ (BSCF)-ceria (CeO_2_)	Zn foil	1.62	0.83 @ 20 mA cm^−2^	131	716	180 cycles, 80 h @ 10 mA cm^−2^	[210]
A,B,C,D	6 M KOH + 0.2 M ZnCl_2_	Pt-Sr(Co_0.8_Fe_0.2_)_0.95_P_0.05_O_3−δ_ (SCFP)/Super P	Zn plate	1.44	0.86 @ 5 mA cm^−2^	122	790.4	240 cycles, 80 h @ 5 mA cm^−2^	[100]
A,B,C,D	6 M KOH + 0.2 M Zn(OAc)_2_	LaMn_0.7_Co_0.3_O_3_ (LMCO)	Zn foil	1.40	0.77 @ 1 mA cm^−2^	35	764	90 cycles, 30 h @ 5 mA cm^−2^	[211]
A,B,C,D	6 M KOH + 0.2 M Zn(OAc)_2_	Ni_3_FeN/V@N-doped graphene	Polished Zn foil	1.52	0.92 @ 10 mA cm^−2^	168	650	150 cycles, 220 h @ 10 mA cm^−2^	[212]
A,C,D	6 M KOH	Fe@N–C-700	Zn plate	1.40	–	220	–	100 cycles, 16.7 h @ 10 mA cm^−2^	[213]
A,C,D	6 M KOH + 0.2 M Zn(CH_3_COO)_2_	NCO@HHPC	Zn plate	1.48	0.73 @ 10 mA cm^−2^	267	767	1460 cycles, 487 h @ 10 mA cm^−2^	[214]
A,C,D	6 M KOH + 0.2 M Zn acetate	Co_5.47_N@N-rGO-750	Zn plate	1.45	0.77 @ 1 mA cm^−2^	121	789	2000 cycles, 330 h @ 1 mA cm^−2^	[215]
A,C,D	6 M KOH + 0.2 M Zn(Ac)_2_	FeNiP/NPCS	Polished Zn plate	1.51	0.58 @ 10 mA cm^−2^	163	603	330 cycles, 110 h @ 10 mA cm^−2^	[216]
A,C,D	6 M KOH/0.2 M Zn(ac)_2_ mixed solution	Ni–Co–S/NSC	Zn plate	1.43	0.73 @ 10 mA cm^−2^	137	829	180 cycles @ 10 mA cm^−2^	[217]
A,C,D	6 M KOH + 0.2 M Zn(CH_3_COO)_2_	GNCNTs-4	Polished Zn foil	1.48	0.76 @ 5 mA cm^−2^	253	801	9000 cycles, 3000 h @ 5 mA cm^−2^	[218]
A,C,D	6.0 M KOH + 0.2 M Zn(Ac)_2_	Ni_1_Co_3_@N–CN	Zn foil	1.446	0.71 @ 5 mA cm^−2^	98.2	721.6	200 h @ 5 mA cm^−2^	[219]
A,C,D	6.0 M KOH + 0.2 M zinc acetate	NCNTM	Zn foil	1.5	0.8 @ 5 mA cm^−2^	220	797	4800 cycles, 1600 h @ 5 mA cm^−2^	[220]
A,C,D	6 M KOH + 0.2 M Zn(Ac)_2_	Co-Co_3_O_4_@NAC	Polished Zn plate	1.45	0.77 @ 10 mA cm^−2^	164	721	35 h @ 10 mA cm^−2^	[97]
A,C,D	6 M KOH + 0.2 M ZnCl_2_	Fe,Co-SA/CS	Polished Zn plate	1.43	0.88 @ 5 mA cm^−2^	86.7	819.6	300 cycles, 100 h @ 5 mA cm^−2^	[221]
A,C,D	6 M KOH + 0.2 M ZnCl_2_	Fe-N_x_-HCS	Polished Zn plate	1.42	1 @ 10 mA cm^−2^	154	422	58 h @ 10 mA cm^−2^	[222]
A,C,D	6.0 mol L^−1^ KOH + 0.2 mol L^−1^ Zn acetate	CoSe_2_@NC loaded on Ni foam	Polished Zn plates	1.48	0.93 @ 10 mA cm^−2^	137.1	751.1	500 cycles @ 10 mA cm^−2^	[223]
A,C,D	6 M KOH + 0.2 M Zn (AC)_2_	CoFe@NC/KB-800	Zn foil	1.351	0.65 @ 2 mA cm^−2^	160	654	600 cycles, 100 h @ 2 mA cm^−2^	[224]
A,C,D	0.2 M Zn(CH_3_COO)_2_ + 6 M KOH	MDPCF-based ZAB	Zn foil	1.48	–	288.8	740	330 h @ 10 mA cm^−2^	[225]
A,C,D	6 M KOH	CoP/NP-HPC	Zn plate	1.4	–	186	–	80 h @ 2 mA cm^−2^	[226]
A,C,D	6 M KOH + 0.2 M zinc acetate	RuCoO_*x*_@Co/N-CNT	Polished Zn plates	1.44	0.79 @ 2 mA cm^−2^	93	788	200 cycles, 34 h @ 2 mA cm^−2^	[227]
A,C,D	6 M KOH + 0.2 M Zn acetate	P,S-CNS	Polished Zn plates	1.51	–	198	830	200 cycles, 40 h @ 2 mA cm^−2^	[228]
A,C,D	6 M KOH + 0.2 M Zn(OAc)_2_	MnCo_2_O_4_@C	Polished Zn plate	1.43	0.72 @ 5 mA cm^−2^	40	–	70 h @ 10 mA cm^−2^	[229]
A,C,D	6 M KOH + 0.2 M Zn(CH_3_COO)_2_	Co/Co_3_O_4_@PGS	Zn plate	1.45	0.96 @ 20 mA cm^−2^	118.27	–	4800 cycles, 800 h @ 10 mA cm^−2^	[136]
A,C,D	6 M KOH + 0.2 M ZnCl_2_	Fe_0.5_Co_0.5_O_*x*_/NrGO	Zn plate	1.43–1.44	0.89 @ 10 mA cm^−2^	86	756	120 h @ 10 mA cm^−2^	[230]
A,C,D	6 M KOH + 0.2 M zinc acetate	Janus NiFe@C@Co CNFs	Polished Zn foil	1.44	0.77 @ 5 mA cm^−2^	130	694	200 h @ 5 mA cm^−2^	[231]
A,C,D	6 M KOH + 0.2 M ZnCl_2_	FeCo@NC-d	Zn plate	1.456	0.79 @ 5 mA cm^−2^	190.2	–	120 cycles @ 5 mA cm^−2^	[232]
A,C,D	6 M KOH + 0.2 M ZnO	CuCo_2_O_4_@CNTs	Zn plate	1.41	0.79 @ 10 mA cm^−2^	–	–	160 cycles, 80 h @ 2 mA cm^−2^	[233]
A,C,D	6 M KOH + 0.2 M Zn(CH_3_COO)_2_	Fe_2_Ni@NC	Zn plate	1.493	0.805 @ 50 mA cm^−2^	126	–	500 cycles @ 10 mA cm^−2^	[234]
A,C,D	KOH/Zn(Ac)_2_	CoNi@CoCN	Zn plate	1.498	–	162.5	773.5	200 cycles	[235]
A,C,D	6 M KOH + 0.2 M Zn(CH_3_COO)_2_	NPSC-Co_2_Fe_1_	Zn foil	1.44	0.96 @ 10 mA cm^−2^	174.6	–	210 cycles, 70 h @ 5 mA cm^−2^	[236]
A,C,D	6 M KOH + 0.2 M ZnCl_2_	CoS_*x*_/Co-NC-800	Zn foil	1.40	0.73 @ 2 mA cm^−2^	103	770.4	450 cycles, 90 h @ 5 mA cm^−2^	[237]
A,C,D	6 M KOH + 0.2 M Zn(OAc)_2_	FeCo–N–C-700	Tailored Zn plate	1.39	–	150	518	360 cycles, 60 h @ 1 mA cm^−2^	[238]

A: Bifunctional oxygen electrocatalyst, B: Perovskite oxides as electrocatalysts, C: Carbon-based electrocatalysts, D: Hybrid/mixed electrocatalysts

## Advanced Form of ZABs

In addition to high energy efficiency, good mechanical properties and flexibility are important for the commercialization of ZABs in wearable, portable, and flexible electronic devices. In practical applications, ZABs must provide stable and satisfactory electrochemical performance under high open-circuit voltages, energy density, power density, cell capacity, and various deformation states such as bending, twisting, and even stretching. Therefore, in addition to the design of the cathode electrocatalyst, anode and solid electrolyte, and separator, a rational and efficient cell configuration also plays an important role in the performance of ZABs. This section describes two recently reported advanced ZAB battery configurations: a mechanical rechargeable battery and a flexible zinc–air battery.

### Mechanically Rechargeable ZABs

Mechanically rechargeable batteries (MR-ZABs) offer an alternative to electrically rechargeable batteries, allowing for the physical replacement or removal of the consumed Zn electrode and electrolyte. These batteries are regarded as primary batteries that can be refurbished and recharged, avoiding the issues of dendritic Zn deposition associated with electrically rechargeable batteries. As a result, simpler unifunctional catalysts that only need to operate in the discharge mode can be used. For these reasons, extensive research efforts have been dedicated to the development of MR-ZABs. For example, Singh et al*.* [20] demonstrated surface tunable spherical cobalt oxide (Co_3_O_4_) NPs distributed over N-doped graphene. The prepared composite (Co_3_O_4_–SP/NGr-24 h) acted as a cathode electrocatalyst for MR-ZABs and exhibited excellent stability and minimal voltage loss at 50 mA cm^−2^. In another report, Kharabe et al*.* [239] proposed hydrothermally synthesized boehmite-phase aluminum oxyhydroxide (AlOOH) nanosheets anchored on N-doped graphene. The prepared composite (AlOOH/NGr) demonstrated an ORR onset potential of around 0.83 V with a half-wave potential of 0.72, combined with remarkable catalytic robustness in an alkaline solution. When utilizing AlOOH/NGr as an air electrode in a primary ZAB, an OCV of 1.27 V, a flat discharge curve at 10 mA cm^−2^, a specific capacity of 720 mAh g^−1^, and a power density of 204 mW cm^−2^ were observed. Additionally, the homemade battery exhibited a long life of over 35 h at 10 mA cm^−2^ after four cycles of mechanical recharging.

**Fig. 12:**
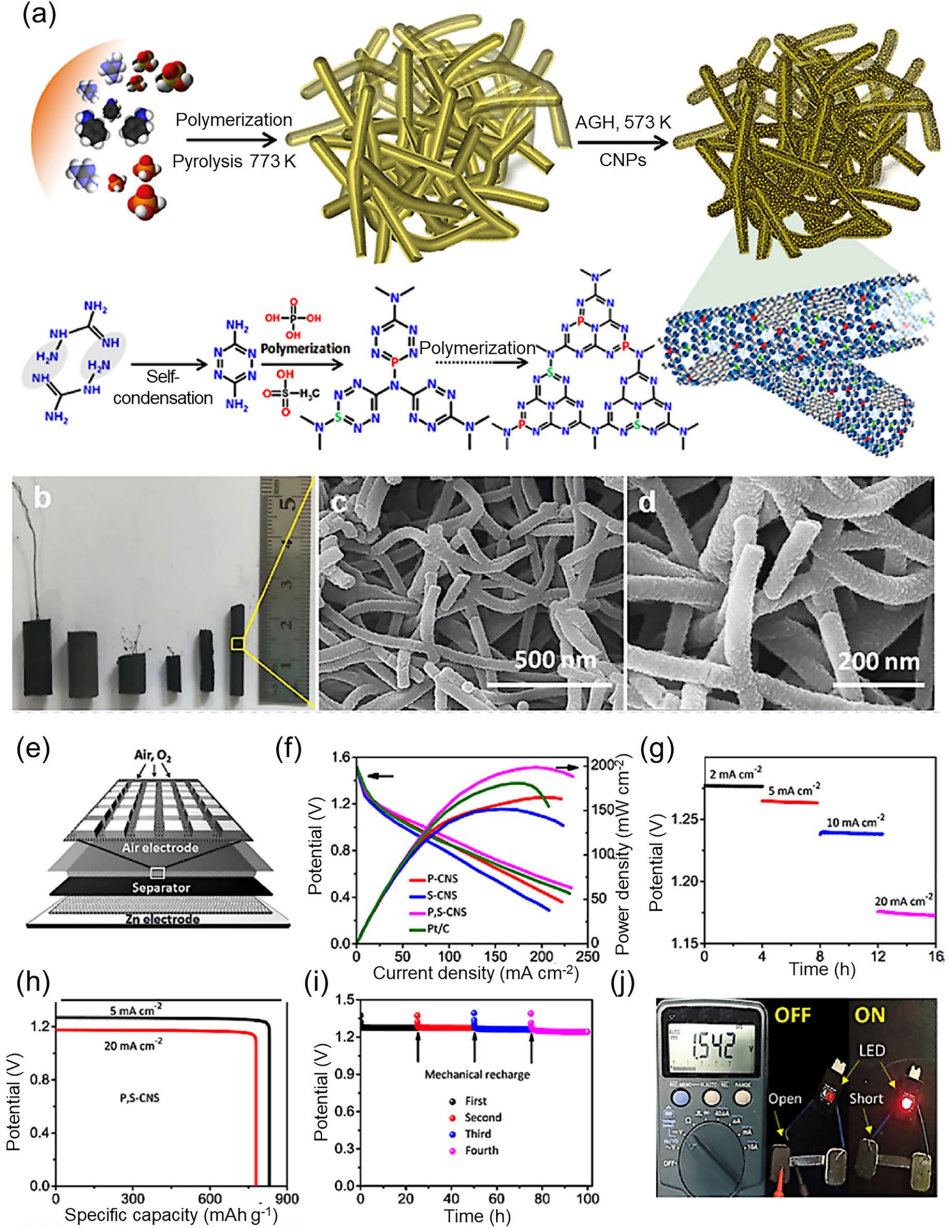
**a** Synthesis process for the sponge-like P,S-CNS catalyst and associated reaction mechanisms. **b** Photograph of the prepared P,S-CNS samples and **c, d** corresponding SEM images. **e** Schematic diagram of the primary ZAB. **f** Polarization curves and calculation of the power densities for primary ZABs constructed using various catalysts. **g** Galvanostatic discharge curves for the primary ZAB using P,S-CNS as the air cathode. **h** Specific capacity of the primary ZAB using P,S-CNS as the ORR catalyst. **i** Stability of the primary ZAB using a P,S-CNS cathode with mechanical recharging. **j** Photograph of LED illumination powered by the proposed ZAB. Reproduced with permission from [228]

Shinde et al*.* [228] proposed a scalable carbon nitride (CN) sponge as an oxygen electrocatalyst for rechargeable ZABs (Fig. 12). The prepared sponge (denoted as P,S-CN) was prepared via pyrolysis followed by polymerization; its possible reaction mechanisms are illustrated in Fig. 12a. Optical images revealed a sponge-like structure associated with diverse dimensions (Fig. 12b), with arbitrarily positioned and entangled 3D hierarchical network of tubular P,S-CNS observed in HRSEM images (Fig. 12c, d). The prepared sample was then used in a primary ZAB as the air cathode (Fig. 12e**)**, with its performance then analyzed based on polarization curves and its power density (Fig. 12f), galvanostatic discharge curves (Fig. 12g), its specific capacity for the ORR process (Fig. 12h), its long-term stability as an MR-ZAB (Fig. 12i), and as the power source for an illuminated LED (Fig. 12j). This 3D P,S-CNS structure led to the fabrication of a primary ZAB with an excellent specific capacity of 830 mA h g^−1^, power density of 198 mW cm^−2^, OCV of 1.51 V, and superior robustness over 210 h after several mechanical recharges. This high performance was attributed to its bifunctional activity due to dual doping and the effective mass/charge transfer.

MR-ZABs can be classified as refuellable or reconstructable cells. Zn plates or cassettes of reconstructable cells can be physically removed and regenerated upon discharge. For smooth operation, a complex industrial facility is required for this type of cell design [16]. The reconstructable cells also face challenges in the reverse reaction at the air cathode, making it difficult to release O_2_ from OH^−^ and water. In addition, usage of expensive Pt catalyst is another bottleneck of this design. In contrast, the battery is recharged with aqueous electrolytes in refuellable cells. Recharging is accomplished simply by exchanging the cassettes. The used Zn anode can be subsequently recycled or refined into Zn. The hydraulic systems can pump alkaline electrolytes through a static bed of Zn particles or circulate the Zn slurry through the battery’s anode compartment, resulting in mitigation of dendritic formation issues at the anode. The refuellable cells can store the electroactive components necessary for battery operation in a tank situated outside the battery structure, which offers greater flexibility in terms of energy and power decoupling. The refuellable cell design of ZAB has less mechanical stress on the electrodes, enabling long-lasting system development for large-scale applications [240]. However, the high manufacturing costs associated with ensuring a stable Zn supply and establishing recharging stations hinder their widespread adoption, despite significant advancements in MR-ZAB technology.

### Flexible Zn–Air Batteries

Smart electronics and flexible devices have been highlighted for use in wearable and portable electronic systems*,* including intelligent bracelets, wearable cell phones, and human-like electronic skin [241–244]. The electrochemical ability and adaptability of each component in these devices is crucial to their realization. A recent trend in the design and fabrication of power sources such as supercapacitors and batteries has been the development of stretchable, flexible, and wearable characteristics [245, 246]. In particular, a range of flexible battery designs has been reported, including stretchable, cable, and bendable types [242, 247–249], with the safety, low cost, environmental friendliness, and high energy density of ZABs particularly suitable for the development of flexible and portable systems [95]. However, the flat, stacked, and rigid designs of conventional ZABs with alkaline electrolytes have restricted their use in flexible and portable applications. Peng and coworkers [38, 250–253] have been pioneers in the development of cable-type structures for use in 1D batteries and supercapacitors, but the potential for versatile ZAB design has been overlooked to date.

**Fig. 13:**
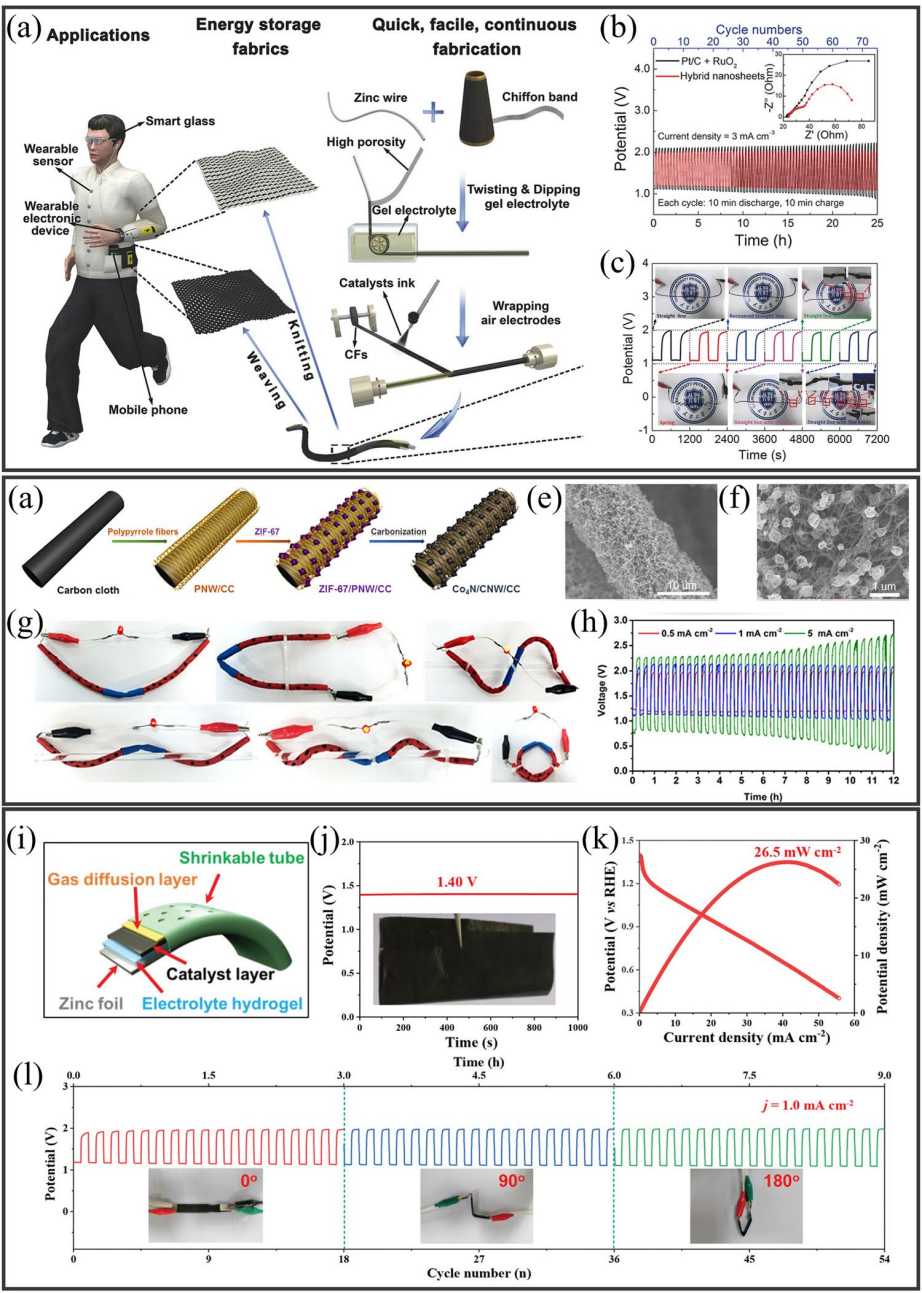
**a** Fabrication process for the air electrode in a fiber-shaped flexible ZAB. **b** Galvanostatic charge–discharge plots for ZABs using different electrocatalysts. **c** Galvanostatic charge–discharge curves for a fiber-shaped flexible ZAB under different deformation conditions. Reproduced with permission from [254]. **d** Fabrication process for a Co_4_N/CNW/CC electrode. **e, f** Low- (10 µm) and high-resolution (1 µm) SEM images for the prepared Co_4_N/CNW/CC electrode. **g** Images of a cable-type flexible ZAB under different twisting or bending conditions. **h** Current density-dependent galvanostatic charge–discharge curves for the cable-type flexible ZAB. Reproduced with permission from [34]. **i** Schematic diagram of a flexible ZAB based on a Co/N@CNTs@CNMF-800 cathode. **j** Measurement of the OCV with the inset showing the flexible electrode. **k** Polarization and power density plots. **l** Cyclic stability of the flexible ZAB under different bending conditions. Reproduced with permission from [255]

The air cathode, which consists of an active material and a porous current collector, is a critical component of assembled ZABs because it is where the ORR and OER occur. Conventionally, carbon paper-based electrodes have been used for ZABs but are a rigid substrate and thus not suitable for flexible devices. Thus, it is necessary to design and develop flexible air electrodes with higher electrochemical performance and flexibility. Li et al*.* [254] demonstrated a flexible air electrode using a spray method to coat a bifunctional catalyst onto carbon fiber (Fig. 13a–c). The bifunctional catalyst consisted of mesoporous Co_3_O_4_ nanosheets (Co_3_O_4_-NSs) and N-doped rGO (N-rGO) and had a uniform morphology. The prepared catalyst demonstrated exceptional ORR and OER activity due to its high surface area, mesoporous framework, and synergy between Co_3_O_4_-NSs and N-rGO. A fiber-based ZAB was constructed using this air cathode (Fig. 13a), which demonstrated excellent cyclic stability and charge–discharge polarization characteristics, outperforming a commercially available Pt/C + RuO_2_ electrode (Fig. 13b). The prepared ZAB also had the ability to sustain severe deformation without the loss of significant electrochemical performance (Fig. 13c), highlighting its potential application in wearable electronics.

Another unique strategy is the use of freestanding air cathodes in which the active material is grown directly on the flexible substrate. This approach favors rapid mass transfer and offers a robust electrode structure. Meng et al*.* [34] recently demonstrated a freestanding cathode using Co_4_N NP-deposited carbon fiber embedded on carbon cloth (CC) (Fig. 13d–f). Due to the presence of a hierarchical network and the combined role of Co_4_N and Co–N–C, the prepared Co_4_N/CNW/CC samples exhibited excellent catalytic activity combined with remarkable stability for the ORR and OER. A flexible ZAB was then constructed using a Co_4_N/CNW/CC electrode. The prepared ZAB demonstrated superior flexibility (Fig. 13g) and remarkable rechargeability (Fig. 13h). This high performance of the flexible ZAB means that it is a promising candidate for use in wearable devices.

**Fig. 14:**
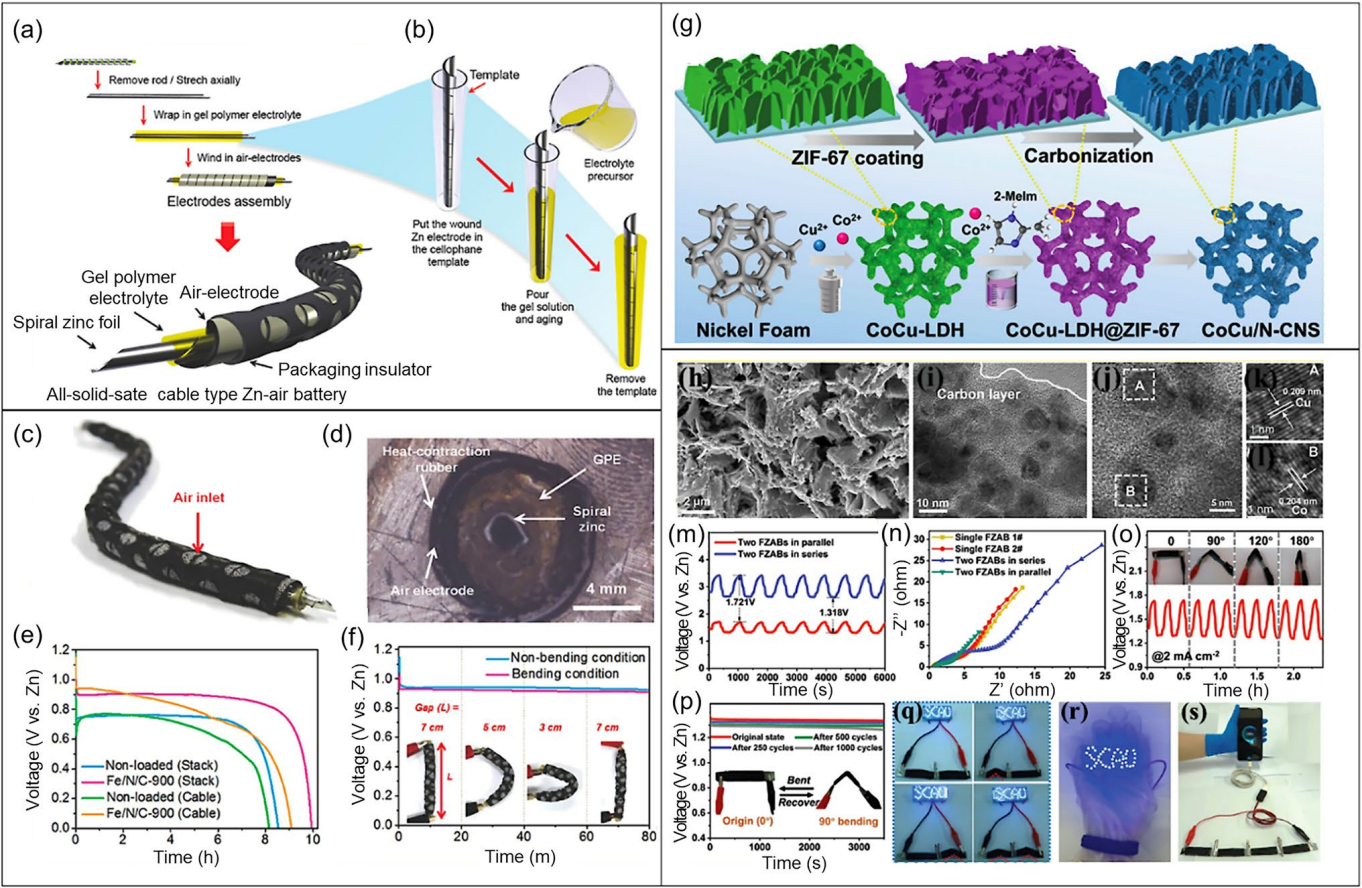
**a** Fabrication process for a solid-state flexible cable-type ZAB. **b** Coating of gelatin-based GPE and KOH (0.1 M) on a spiral zinc anode. **c** Photograph of a prototype flexible cable-type ZAB. **d** Cross-sectional optical microscope image of the cable-type ZAB. **e** Discharge curves for cable-type and stacked ZABs with/without the Fe/N/C electrocatalyst measured at a current density of 0.1 mA cm^−2^. **f** Discharge curve measurements for a flexible cable-type ZAB under different bending conditions. Reproduced with permission from [36]. **g** Fabrication process for the synthesis of the electrocatalyst CoCu/N-CNS-*x* (*x* = 1, 2, 3) over nickel foam. **h** SEM image (2 µm). **i** TEM image (10 nm). **j-l** HRTEM images (5 nm and 1 nm) showing the presence of Cu and Co in the prepared electrocatalyst. **m** Measurement of charge–discharge plots for two flexible ZABs connected in parallel or series and **n** corresponding Nyquist plots. **o** Charge–discharge measurements for the flexible ZAB under different bending conditions at 2 mA cm.^−2^. **p** Voltage measurements for the flexible ZAB after bending and recovery. **q** Illumination of an LED powered by two flexible ZABs connected in series under various bending settings. **r** Image of a wearable bracelet containing a flexible solid-state ZAB used to power the LED screen. **s** Charging of a mobile phone using four ZABs in series. Reproduced with permission from [256]

Two strategies are commonly used to enhance mechanical flexibility in wearable electronics: (1) adhesive-assisted loading of the active material on a flexible substrate, and (2) direct growth of the active material on a flexible substrate, eliminating the need for adhesives or additives. The latter approach, which is cost-effective and scalable, is particularly well suited for flexible electrode design. Liu et al*.* [255] proposed an easy and scalable method to construct high-quality bifunctional freestanding air electrodes for flexible ZABs (Fig. 13i–l). The solid-state flexible ZAB design consisted of an anode of Zn foil, the prepared electrocatalyst (Co/N@CNTs@CNMF-800) as the cathode, and KOH/PVA as a gel electrolyte (Fig. 13i). The flexible ZAB demonstrated an OCV of 1.40 V (Fig. 13j) and power density of 26.5 mW cm^−2^ (Fig. 13k). In addition, the flexible ZAB exhibited remarkable stability with bending angles of 90° and 180° (Fig. 13l). The flexible ZAB could be further exploited for use in wearable systems. Park et al*.* [38] reported an all-solid-state cable-type flexible ZAB. Their system consisted of a spiral-type Zn anode, a gel-type polymer electrolyte, and an inexpensive Fe/N/C electrocatalyst-based air cathode (Fig. 14a–f). Figure 14a, b presents the fabrication process for the flexible cable-type ZAB, while a photographic image of the ZAB is displayed in Fig. 14c, showing holes that act as air inlets for the O_2_ used in the electrocatalytic reactions. Figure 14d presents an optical micrograph for a cross section of the flexible cable-type ZAB. To test the electrocatalytic performance of the proposed ZAB, both stacked and cable-type ZABs were fabricated using a gelatin-based GPE (GGPE; Fig. 14e). Interestingly, both the stacked and cable-type ZABs with the Fe/N/C-900 electrocatalyst produced a higher performance than those where the electrocatalyst was absent. The device was also tested for voltage stability under bending/non-bending conditions (Fig. 14f). No significant voltage loss was observed, confirming that the prepared electrocatalyst was suitable for use in wearable applications. Kuang et al*.* [256] also prepared an all-solid-state flexible ZAB based on the unique electrocatalyst CoCu/N-CNS as the cathode (Fig. 14g–s). The proposed strategy allowed for the direct growth of the prepared electrocatalyst over Ni foam. The various steps involved are shown in Fig. 14g. The prepared electrocatalyst contained tightly bound nanosheets with abundant embedded NPs (Fig. 14h), with the NPs clearly seen in TEM images (Fig. 14i–l). The lattice spacing as assessed using TEM analysis indicated the presence of both Co and Cu in the carbon nanosheet framework. To test the flexibility and electrocatalytic performance of the proposed cathode, the change in the voltage of a ZAB was tested (Fig. 14m), while Nyquist plots were obtained to measure the resistance for ZABs connected in series and parallel (Fig. 14n). In addition, the flexibility and stability of the ZAB meant that there was no significant change in the voltage under different bending conditions (Fig. 14o, p). An LED light, wearable bracelet, and mobile phone were also powered using the proposed ZAB, producing an excellent illumination performance (Fig. 14q–s). The prepared system thus offers a facile device design and has the potential for use in next-generation wearable and portable energy storage devices.

## Conclusions and Outlook

Zinc–air batteries (ZABs) have a higher theoretical energy density (1218 Wh kg^−1^) compared to LIBs, making them more energy-efficient in a form factor and thereby enabling in a lighter and cheaper design. This suggests a promising avenue for substantial progress in the realm of ZABs for portable and flexible versions that can easily integrate into wearable devices, such as cell phones, electronic skins, and intelligent bracelets. Moreover, ZABs are a much more promising alternative, from the environmentally friendly viewpoint, because they utilize oxygen from the air as a reactant, which reduces the need for heavy metal components within the battery. ZABs do not rely on scarce metal resources, making them a more sustainable option over LIBs. However, ZABs currently face challenges in achieving their full theoretical energy density and suffer from limitations related to electrode degradation and electrolyte management. Also, conventional ZAB designs with rigid and stacked structures using aqueous alkaline electrolytes do not align with the requisites of portability and flexibility. Exploration of the wearability, including mechanical deformation (bending, twisting, folding, stretching, and compressing), flexibility, stretchability, thermal conductivity, permeability of air and water molecules, is a paramount significance to accelerate the rapidly evolving of applications. Hence, future research endeavors must be directed toward the development of solid-state or quasi-solid-state polymer-based electrolytes, possessing both elevated ionic conductivity and mechanical strength. In this context, we tackle these concerns and deploy efficient strategies to fully actualize the benefits offered by ZAB technology.

(1) Maximizing energy density through elevated catalytic activity: Limited energy density remains a significant challenge for ZABs because practical applications have been struggled to achieve the potential of high theoretical energy density. The actual energy density of ZABs currently falls below that of other battery technologies, such as LIBs. The reactions of ORR (oxygen reduction reaction) and OER (oxygen evolution reaction) play an important role in overcoming this challenge. ORR requires effective contact between the electrocatalyst, air electrode, and electrolyte due to its gas-consuming nature. Conversely, for OER, prompt separation of the generated oxygen from the interface between electrocatalyst and air electrode is essential. This disparity necessitates different hydrophobicity levels for catalysts and air electrodes in OER and ORR reactions.

The catalytic activity of catalysts is also crucial for efficient electrochemical processes, and two main approaches enhance intrinsic activity: heteroatom (N, O, S, B, P, etc.) doping and porous structure control. Heteroatom doping induces charge redistribution and alters electronic structure, reducing adsorption energy of oxygen-containing species and thereby enhancing catalytic activity. Pore size engineering of the electrode materials increases the density of active sites. Macro-/mesopores facilitate the mass transfer allowing reactants to easily access the active centers and promote the diffusion of oxygen and reactants, whereas micropores provide a large specific surface area supporting a high degree of dispersion of active sites. To further enhance the utilization of active sites while preventing nanoparticle agglomeration, various strategies, including core–shell design, space confinement, and hierarchical structures, can be employed. The effective integration of metal active species with low-dimensional carbonaceous materials, such as carbon nanotubes (CNT) and graphene, has emerged as a promising approach to develop hybrid electrocatalysts characterized by high electrocatalytic activity, rapid electron transfer rates, superior selectivity, and satisfactory stability. The presence of π-conjugated conductive ligands and unbound electrons dissociated from the *sp*^2^ graphitic structure ensures fast charge transport during redox reactions, which greatly enhances electrocatalytic activity. These approaches also ensure the uniform loading of metal catalytic active sites onto functionalized carbon supports, leading to a strong coupling synergetic effect.

(2) Long-term stability/durability: The utilization of zinc gives rise to a noteworthy consideration. Firstly, long-term performance and practical usability are impeded by challenges, such as enduring permanent damage from corrosive by-products, irreversible ZnO deposition, and the formation of zinc dendrites. The presence of subsequent by-products, such as hydrogen peroxide, triggers avalanche damage of the air electrode, leading to a substantial decrease in the stability of rechargeable ZAB. Moreover, the unresolved electrochemical oxidation behavior of the OER for carbon-based electrodes during charging results in decreased electrical conductivity and hydrophobicity after multiple deep charge and discharge cycles, further compromising the battery's stability.

Second, the deposition of ZnO on the zinc anode poses another challenge as it obstructs further reactions between electrolyte and electrode structure, affecting the structural integrity of the electrode. Excessive ZnO obstructs the pores of the air electrode, impeding the rapid transport of gas and ion, reducing the active reaction area and stability. The stability of the ZABs is also affected by uneven distribution of active components during charging process of zinc anode, due to the liquid-phase mass transfer resistance. This results in higher deposition rates of active components on protruding regions of the zinc electrode surface, causing to the formation of zinc dendrites and altering the electrode’s shape. These dendrites pose a grave challenge to the overall stability and functionality of the ZABs.

Last, the sensitivity of zinc to moisture is a significant concern, leading to self-discharge reactions when exposed to humid environments, ultimately reducing the battery's shelf life and overall performance. The air electrode is also susceptible to degradation over time due to the complex ORR occurring on its surface. Continuous exposure to harsh electrochemical conditions during charge/discharge cycles leads to the progressive deterioration of the electrode's catalytic activity and structural integrity. To address this issues, robust encapsulation techniques and/or moisture-resistant materials are essential to enhance the reliability of ZABs.

Besides, significant reduction in performance stability and durability is observed upon deactivating the catalysts in ZABs. Conventional cell fabrication methods using casting or coating with polymeric binders are often considered laborious and time-consuming. In this context, self-supported electrocatalysts directly grown on conductive substrates, such as carbon cloth, Ni foam, and stainless steel (SS) mesh, have emerged as compelling choices. Numerous approaches are centered around electrode design enhancements aimed at bolstering this stability. Direct growth (in situ) of highly active catalysts on corrosion-resistant conductive substrates is an efficient strategy to inhibit cycle corrosion effects in high-efficiency air electrodes. It can also offer bendability, portability, crumpled morphology, and lightweight nature. The integration of catalytic materials with conductive substrates creates opportunities to advance electrocatalytic technologies and presents an attractive prospect for catalysis research and development.

(3) Advanced ionic liquid or (quasi- or all-) solid-state electrolytes: The choice of electrolyte is vital to ensure ZABs’ enduring functionality, acting as the conduit for ion migration during charging/discharging, impacting ohmic resistance of batteries. Like any other batteries, evaporation and leakage of electrolytes are also critical issues affecting the device performance, safety, and lifetime of ZABs. Conventional alkaline electrolytes in primary ZABs suffer from zincate precipitation and carbonation, limiting their cycle life. The vulnerability to atmospheric CO_2_ leads to carbonate formation, reducing conductivity and hindering air diffusion. This increases polarization and shortens battery lifespan. Although many efforts have been made to mitigate carbonate formation through physicochemical adsorption of CO_2_, such approaches may result in considerable material and management costs.

Ionic liquids are potential alternatives to conventional aqueous alkaline electrolytes, offering benefits such as reduced electrolyte evaporation, resistance to hydrogen evolution and CO_2_ poisoning, and improved battery efficiency and cycle life. Additionally, electrolyte additives, such as zinc acetate and citric acid, can enhance the performance of ZABs by improving the shape change of the zinc electrode. Introducing inorganic or organic additives into electrolytes helps mitigate dendrite formation and irregularities on electrode surfaces. For example, the addition of polyethylene glycol (PEG) as an electrolyte additive has been demonstrated to effectively mitigate the kinetics of zinc electrodeposition and inhibit the growth of zinc dendrites.

Quasi- or all-solid-state electrolytes, serving dual roles as ion conductors and separators, simplify battery design and manufacturing processes. This approach effectively tackles issues tied to highly active zinc electrodes and aqueous electrolytes, mitigating concerns such as corrosion, passivation, and dendrite growth. This is achieved through the advantageous attributes of semisolid electrolytes, such as their restricted water content and high elastic modulus, which facilitate effective alleviation of these challenges. Employing semisolid electrolyte systems offers a promising avenue to address challenges associated with interactions, enhancing the performance and stability of zinc-based electrochemical systems.

Research is ongoing to explore novel electrolyte formulations for enhanced performance and cyclability. The water-in-salt (WIS) electrolytes are a recent invention to prohibit anodic HER and widen the potential window [257]. The high salt concentration electrolytes could be an effective strategy to address issues like HER and dendrite growth at the anode side. Dong et al*.* reported a highly concentrated aqueous electrolyte containing zinc acetate with unprecedented solubility (up to 23 m) by using the hydrotropic agents that transform the acetate anion ligands into a hydrophilic coordination structure. The hydrotropic agents, including potassium acetate, urea, and acetamide, are effective in constructing highly concentrated zinc acetate electrolytes which retain 70% of their initial capacity after 4,000 cycles on Zn//pyrene-4,5,9,10-tetraone full cell of ZABs [258]. Further, efforts toward improving the electrolyte–electrode interfacial properties must be emphasized when replacing the incumbent liquid electrolytes with versatile solid-state alternatives.

(4) Portable and flexible ZABs: Recent developments in portable and flexible ZABs hold great promise for wearable devices, such as wearable cell phones, human-like electronic skins, and intelligent bracelets. The fabrication of flexible ZABs necessitates the integration of flexible current collectors, electrolyte membranes, and encapsulating materials. Careful design of each component is essential to maintain stable electrochemical performance, even under deformation. Moreover, ensuring structural and mechanical stability of the fabricated electrodes is vital for long-term and cyclic utilization. As aforementioned, a key challenge for future research is fabricating all- or quasi-solid-state electrolytes, exhibiting both high ionic conductivity and mechanical robustness. The utilization of binder-free self-supported air electrodes excels in meeting the demanding requirements of flexible devices. These advantages include simplifying the design and fabrication of air electrodes, allowing for practical and scalable preparation of self-supported electrodes. Thus, enhancing the interaction between active materials and substrates through judicious optimization of preparation conditions emerges as a critical aspect to be pursued in order to achieve the desired objectives in the development of flexible ZABs.
